# Synthesis of Novel Tryptamine Derivatives and Their Biological Activity as Antitumor Agents

**DOI:** 10.3390/molecules26030683

**Published:** 2021-01-28

**Authors:** Giorgia Simonetti, Carla Boga, Joseph Durante, Gabriele Micheletti, Dario Telese, Paolo Caruana, Andrea Ghelli Luserna di Rorà, Fabio Mantellini, Samantha Bruno, Giovanni Martinelli, Natalia Calonghi

**Affiliations:** 1 Biosciences Laboratory, IRCCS Istituto Romagnolo per lo Studio dei Tumori “Dino Amadori”—IRST S.r.l., 47014 Meldola (FC), Italy; giorgia.simonetti@irst.emr.it (G.S.); andrea.ghellilusernadirora@irst.emr.it (A.G.L.d.R.); giovanni.martinelli@irst.emr.it (G.M.); 2 Department of Industrial Chemistry “Toso Montanari”, Alma Mater Studiorum—Università di Bologna, Viale del Risorgimento 4, 40136 Bologna, Italy; gabriele.micheletti3@unibo.it (G.M.); dario.telese2@unibo.it (D.T.); paolo.caruana@gidi.it (P.C.); 3 Department of Biomolecular Sciences, University of Urbino “Carlo Bo”, Via I Maggetti 24, 61029 Urbino (PU), Italy; jdurante6@gmail.com (J.D.); fabio.mantellini@uniurb.it (F.M.); 4 Department of Experimental, Diagnostic and Specialty Medicine, Institute of Hematology and Medical Oncology “L. and A. Seràgnoli”, University of Bologna, 40138 Bologna, Italy; samantha.bruno2@unibo.it; 5 Department of Pharmacy and Biotechnology, University of Bologna, 40121 Bologna, Italy

**Keywords:** cancer, tryptamine, leukemias, chloral, pyrimidine

## Abstract

We synthesized five novel tryptamine derivatives characterized by the presence of an azelayl chain or of a 1,1,1-trichloroethyl group, in turn connected to another heterocyclic scaffold. The combination of tryptamin-, 1,1,1-trichloroethyl- and 2-aminopyrimidinyl- moieties produced compound **9** identified as the most active compound in hematological cancer cell lines (IC_50_ = 0.57–65.32 μM). Moreover, keeping constant the presence of the tryptaminic scaffold and binding it to the azelayl moiety, the compounds maintain biological activity. Compound **13** is still active against hematological cancer cell lines and shows a selective effect only on HT29 cells (IC_50_ = 0.006 µM) among solid tumor models. Compound **14** loses activity on all leukemic lines, while showing a high level of toxicity on all solid tumor lines tested (IC_50_ 0.0015–0.469 µM).

## 1. Introduction

**Figure 1:**
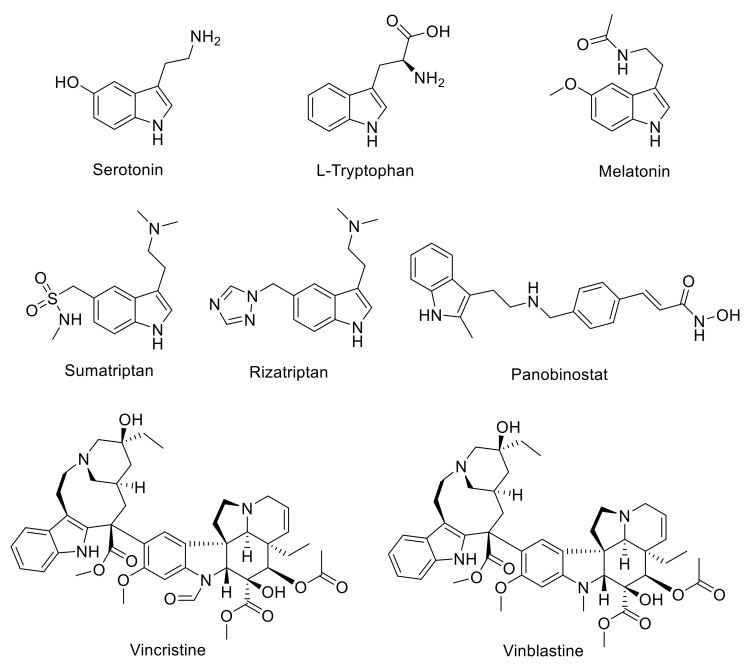
Natural products and pharmaceuticals containing tryptamine subunit.

The indole nucleus (**1**) belongs to many natural heterocyclic compounds and is of interest in many applied fields, such as industrial, agricultural and medicinal chemistry [1]. In many natural molecules and synthetic or semi-synthetic drugs this scaffold represents a pharmacophoric group fundamental for the interaction with receptors. Among the plethora of indole derivatives present in the animal and vegetal realm, a great importance has been recognized to tryptamine (**2**). The tryptamine is a biosynthetic precursor of many natural alkaloids [2,3] and is frequently used as chemical building block in the total synthesis of biologically active and pharmaceutically important compounds [4,5]. In Figure 1 are shown some examples of natural tryptamine derivatives with important biological roles such as l-tryptophan, serotonin, melatonin, besides the tryptane drugs sumatripan, rizatripan and the anticancer agents vincristine, vinblastine and panobinostat.

**Figure 2:**
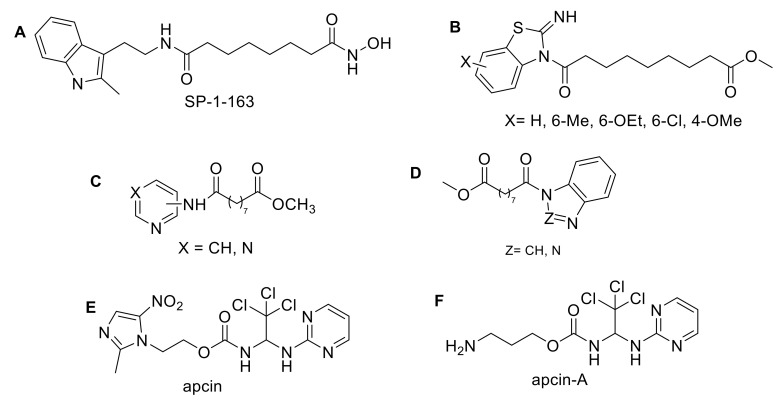
Biologically active compounds bearing (**A**) indole-, (**B**) thiazole-, (**D**) triazole-, (**C**) pyridinyl- and (**E**,**F**) pyrimidinyl-scaffolds.

The latter is active against multiple myeloma with a mechanism involving inhibition of histone deacetylase (HDAC-6) [6], as well as recently patented [7] structures containing an aliphatic chain ending with a hydroxamic acid and an amide group involving a tryptamine subunit (compound **SP-1-163**, Figure 2A). 

Recently, we synthesized a series of compounds with structural analogy with compound **SP-1-163** (Figure 2A). We prepared structural hybrids between azelaic acid methyl ester and several amino-aza-heterocycles (Figure 2B,D). The azelayl moiety recalls part of 9-hydroxystearic acid, a cellular lipid showing antiproliferative activity toward cancer cells with HDAC as the molecular target [8,9,10,11,12,13,14,15,16]. Actually, we found that some derivatives belonging to series in Figure 2B–D behave as selective histone deacetylase inhibitors (HDACi): benzothiazole-based series (Figure 2B) is active against HT29 human colon cancer cells line [17] whereas, among the series containing pyridinyl (or piperidinyl, benzimidazolyl, benzotriazolyl) groups (Figure 2C,D), two aminopyrimidinyl and the benzimidazolyl derivatives showed specific activity on osteosarcoma cells [18]. The two latter moieties, aminopyrimidyl- and imidazolyl- one are also present in Apcin, (Figure 2E) a small molecule that blocks the interaction between APC/C and Cdc20 [19]. A recently published patent reported the synthesis and biological tests of many derivatives of Apcin-A (Figure 2F) [20]. However, in none of the above cited articles, the tryptamine moiety is present. 

Due to the importance of both, trichloromethyl and aminopyrimidyl group, in influencing the biological activity of some above cited compounds, we centred our attention towards the synthesis of novel tryptamine derivatives bearing both these structural motifs and structural hybrids connecting one tryptamine moiety an azelayl one. Herein we report the synthesis and the results obtained on the biological activity of the novel compounds towards a panel of solid and hematological cancer cell lines.

## 2. Results and Discussion

### 2.1. Chemistry

#### 2.1.1. Synthesis of Ureido Derivatives **4** and **5**, Bearing Tryptamine and Trichloromethyl Moieties

**Scheme 1:**
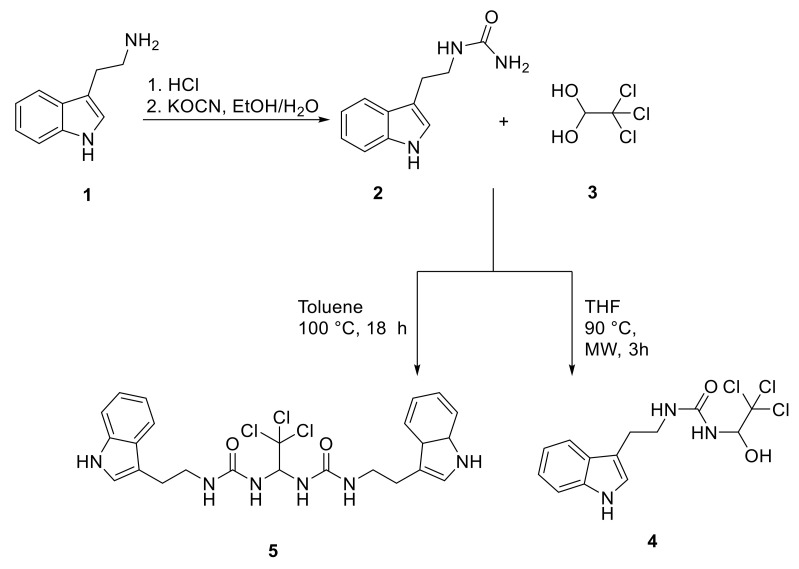
Synthetic pathway to obtain compounds **4** and **5**.

First, we synthesized compounds **4** and **5**, bearing both tryptamine and trichloromethyl moieties. The reaction of tryptamine with potassium cyanate gave the ureido derivative **2** that, by reaction with chloral hydrate under different experimental conditions, produced compound **4** or compound **5** bearing, respectively, one and two tryptamine moieties (Scheme 1). Compounds **4** and **5** have been obtained, after purification by column chromatography on silica gel, in 93% and 50% yield, respectively and have been fully characterized.

#### 2.1.2. Synthesis of *N*-(2-(1*H*-indol-3-yl)ethyl)-2,2,2-trichloro-*N*′-(pyrimidin-2yl)etan-1,1-diamine (**9**)

**Scheme 2:**
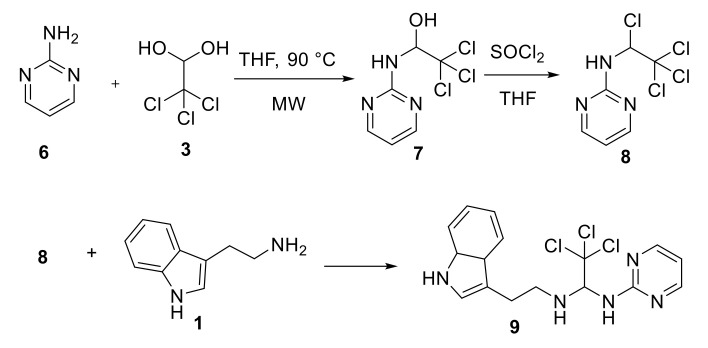
Synthetic route to compound **9**.

Compound **9**, contemporarily bearing tryptamine, pyrimidine and trichloromethyl moieties, was obtained through the multistep procedure shown in Scheme 2. 

2-Aminopyrimidine (**6**) was reacted with chloral hydrate (**3**) in THF under microwaves irradiation giving the hemiaminal (**7**) in 84% yield. The latter was subjected to chlorination by treatment with SOCl_2_ at room temperature in anhydrous THF and under nitrogen flow, in order to remove hydrochloric acid produced during the reaction. Intermediate compound **8** was then reacted without purification with tryptamine (**1**) and the reaction course was monitored by ^1^H NMR of a sample of concentrated crude reaction mixture until to reveal a conversion of about 80%. Compound **9** was recovered, after chromatography on neutral alumina, with purity > 99% even if in only 23% yield; the reason of the low yield might be due to the decomposition of the product during the purification step. We tried also the reaction between compounds **8** and **2** but no product was detected, probably because of the low nucleophilicity of the nitrogen amide atom of compound **2**. Compound **9** was subjected to biological essays, as well as compound **7**, chosen with the aim to compare their activities. 

#### 2.1.3. Synthesis of Hybrids Containing Tryptamine and Azelaoyl Moieties

Based on literature reports on the biological activity of compounds bearing different heterocyclic moieties bound to an azelayl chain through an amide bond [17,18], we planned to synthesize compounds **13** and **14**, bearing a tryptamine group.

**Scheme 3:**
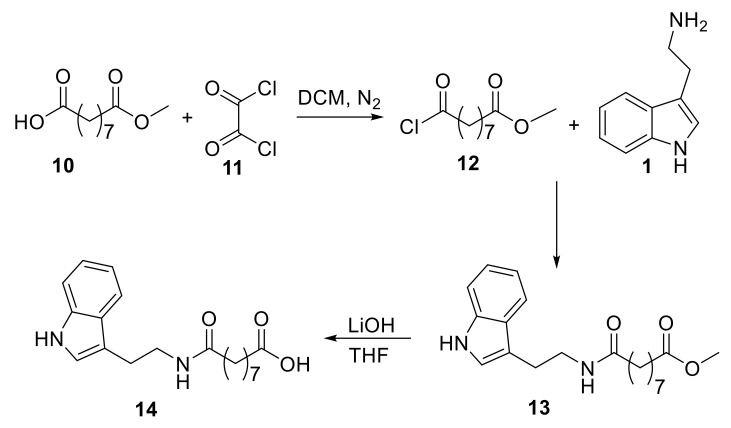
Synthesis of compounds **13** and **14**.

The synthetic approach, shown in Scheme 3, is based on the formation of the amide (**13**) under Schotten-Bauman like conditions. Thus, we first prepared the acyl chloride derivative (**12**) by reaction between the commercially available azelaic acid monomethyl ester (**10**) and oxalyl chloride (**11**). Then, we reacted this activated azelaoyl moiety with tryptamine. The ester bond of compound **13** was further selectively and almost quantitatively hydrolysed to free acid (**14**) in order to check the biological activity of both, the acid and the ester. The novel compounds have been purified and fully characterized. 

### 2.2. Biological Activity 

#### Effect of Compounds on Cells Viability

To investigate whether the compounds have an antitumor activity, we performed in vitro preclinical assays. In vitro growth inhibitory concentration was determined by incubating the cells with increasing concentrations (0.01–250 μM) of the compounds for 24 h. Data obtained from cell growth assays were elaborated to assess the concentration of each compound required for 50% inhibition of cell viability (IC_50_) and the results are shown in Table 1. All the newly synthesized compounds showed a biological activity. However, we observed striking differences in the models response.

Compound **9**, that is characterized by 2-aminopyrimidyl- and trichloroethyl- moieties, similarly to those in Apcin, was active against 3 out of 4 hematological models (MV-4-11, REH and, in particular, the T-cell acute lymphoblastic leukemia cell line Jurkat 6). However, this compound showed poor biological activity when tested in solid tumor models. Indeed, it was able to reduce cell growth only in the osteosarcoma U2OS cell line at high doses (IC_50_ = 235 µM). 

Apcin interferes with the biological activity of Cdc20, that functions as an oncogenic factor in tumorigenesis: it is upregulated across several cancer types, including ovarian/uterine, colon and colorectal cancer, oral squamous cell carcinoma [21] and in aneuploid compared with euploid acute myeloid leukemia [22]. Moreover, its overexpression is associated with poor prognosis in colorectal cancer [23], oral squamous cell carcinoma [24] and serous epithelial ovarian cancer [25], among the tumor types analyzed in the present manuscript. The binding site of Apcin on Cdc20 has been identified [19] and a study on SAR has revealed that the pyrimidine ring and aminal nitrogens, together with the hydrophobic trichloromethyl group, are important structural motifs; in contrast, the elimination of the nitro-imidazole moiety produces little effect. Apcin showed preclinical activity against multiple myeloma [26], prostate cancer [27], glioblastoma [28] and osteosarcoma [29] cells. Very recently, during the current investigation, the synthesis and the in vitro evaluation in a panel of solid tumor cells [MCF-7, A375, A549, HepG2, Hela, Ovcar-3 and Caov-3) of a series of 2,2,2-trichloro-1-aryl carbamate derivatives in which Apcin was modified by changing the pyrimidinyl or the 1-(2-methoxyethyl)-2-methyl-5-nitro-1H-imidazolyl moiety, has been reported [30]. Few studies are currently available on acute leukemias [31]. Withaferin A, a compound inducing degradation of the Mad2-Cdc20 complex was shown to reduce cell viability in acute myeloid and lymphoblastic models [32,33,34]. 

Since compound **9** showed a selective activity against leukemic cell lines, we then investigated whether the tryptaminic and 2-aminopyrimidyl moieties of compound **9** were effective against solid tumor models, when tested separately. We observed that the trichloro-1-(pyrimidin-2-ylamino)ethyl group (compound **7**) maintained some activity against Jurkat 6 cells and was more active against U2OS cells, while solely the presence of tryptaminyl and trichloroethyl groups (connected through an ureido moiety) (as in compound **4**), produced the total loss of activity against hematological models, while being effective against U2OS and the colon cancer cell line HT29. Moreover, the dimeric form of compound **4**, with symmetry with respect to the trichloroethyl group, (compound **5**) showed a further activity reduction, being able to reduce cell growth in a single cell line and at very high doses that hamper any preclinical development. Overall, these data suggest that both the triptaminyl and the pyrimidinyl moieties connected through the trichloroehtyl group (compound **9**) are relevant to the compound effectiveness against acute leukemia models, while showing a modest and selective activity in solid cancers. 

Therefore, we sought to identify novel compounds able to target solid tumors. Keeping constant the presence of the tryptaminic scaffold and binding it to the azelayl moiety (compound **13**), we maintained a biological activity against acute myeloid and B-cell acute lymphoblastic leukemia cell lines and observed a selective effect in HT29 cells, against solid cancer models. Conversely, solid tumor models displayed a specific vulnerability towards compound **14** (the acid free form of the ester of compound **13**), that was not effective against hematological cell lines. Of note, compounds **13** and **14** were both active against HT29 cells, in line with previous observations on similar compounds [17]. Moreover, these compounds recall the structure of HDAC inhibitors, that showed modest efficacy as single agent in acute leukemias [35,36].

## 3. Materials and Methods

### 3.1. Chemical Syntheses

The reagents used, unless stated otherwise, were purchased from Sigma-Aldrich (Milan, Italy). CH_2_Cl_2_ was dried by distillation over P_2_O_5_. Anhydrous THF was freshy distilled over sodium benzophenone ketyl. Chromatographic purifications (FC) were carried out on glass columns packed with silica gel Geduran Si 60, 0.063–0.200 mm, (Sigma-Aldrich, Milan, Italy) or neutral alumina (Brockmann Grade 58 angstroms, Alfa Aesar, Kandel Germany) at medium pressure. Thin-layer chromatography (TLC) was performed on silica gel 60 F254-coated aluminum foils (Fluka, Buchs, Switzerland) or neutral alumina coated plates (Polygram Alox N/UV_254_, Macherey-Nagel, Oensingen, Switzerland).

The nuclear magnetic resonance spectra were recorded at 25 °C on Varian spectrometers Mercury 400 or Inova 600 (Varian, Palo Alto, CA, USA) operating at 400 or 600 MHz (for ^1^H-NMR) and 100.56 or 150.80 MHz (for ^13^C-NMR), respectively. Signal multiplicities were established by DEPT-135 experiments. Chemical shifts were measured in δ (ppm) with reference to the solvent (DMSO-d_6_: δ = 2.50 ppm for ^1^H-NMR and δ = 39.5 for ^13^C NMR; CD_3_CN: δ = 1.96 ppm for ^1^H NMR and δ = 118.26 ppm for ^13^C-NMR; CDCl_3_: δ = 7.26 ppm for ^1^H-NMR and δ = 77.0 ppm for ^13^C-NMR). *J*-values are given in Hz. Electrospray ionization (ESI)-MS spectra and ESI high-resolution HRMS spectra were recorded using Waters ZQ 4000 and Xevo instrument, respectively (Waters Corporation, Milford, MA, USA). IR spectra were recorded using a Fourier transform spectrophotometer PerkinElmer FT-IR spectrometer Spectrum Two in the 4000−400 cm^−1^ wavelength range, using an Universal ATR accessory (Perkin Elmer, Waltham, MA, USA). Melting points (m.p.) were measured on a Büchi 535 apparatus (Büchi, Flawil, Switzerland) and are uncorrected. Methyl 9-Chloro-9-oxononanoate (**12**) was prepared as previously described [17,18]. GC-MS analyses were recorded with a Agilent 6890 Series (Agilent, Santa Clara, CA, USA) gas chromatograph interfaced with a Agilent 5973 Network quadrupole mass selective detector (injection temperature: 250 °C; oven temperature was programmed as follows: 60 °C for 2 min, increased up to 260 °C at the rate of 20°/min, followed by 260 °C for 20 min; the carrier gas was helium, used at a flow rate of 1 mL/min; the transfer line temperature was 280 °C; the ionization was obtained by electron impact (EI); the acquisition range was 50–500 *m*/*z*). Bulb to bulb distillation was carried out using a Buchi GKR-50 apparatus (Büchi Flawil, Switzerland). Reactions under microwave irradiation were carried out using a Milestone Start Synth (Milestone Inc, Shelton, CT, USA) apparatus at 300 W. Copies of ^1^H-NMR, ^13^C-NMR and ESI-Ms spectra of compounds **4**, **5**, **7**, **8**, **9**, **13**, and **14** are reported in Supplementary Materials.

#### 3.1.1. 1-(2-(1*H*-Indol-3-yl)ethyl)urea (Compound **2**)

Tryptamine (compound **1**, 2 g, 0.0125 mol) was introduced in a round bottom flask immersed in a water/ice bath. After 5 min, 37% aq. HCl (1.24 mL, 0.015 mol) was added and the mixture was magnetically stirred for 10 min. Ethanol (10 mL) was added and the system was immersed in an oil bath and heated at reflux for 5 min. To the solution, allowed to stand until to reach the room temperature, a solution of KOCN (1,21 g, 0.015 mol) in H_2_O (10 mL) was added. The resulting mixture was stirred at room temperature overnight then concentrated in vacuo. The residue was purified by flash column chromatography on silica gel (eluent: 8/2 DCM/MeOH) to afford compound **2** (70% yield) as a pale yellow solid whose chemico physical data are in agreement with those reported in the literature. Ref. [37] m.p.: 140.5–142.0 °C (Lit. [37]: 140–142 °C). Below we report the ^1^H-NMR spectrum, never published in CD_3_OD: (400 MHz, CD_3_OD, 25 °C) δ, ppm = 7.56 (dt, *J* = 8.0 Hz, *J*= 1.0 Hz, 1 H), 7.32 (dt, *J*= 8.0 Hz, *J*= 0.9 Hz, 1 H), 7.08 (ddd, *J* = 8.0 Hz, *J* = 7.1 Hz *J*= 1.3 Hz 1 H), 7.07 (br.s., 1 H), 6.9 (ddd, *J*= 8.0 Hz, *J*= 7.1 Hz*, J* = 1.1 Hz 1 H), 3.41 (t, *J* = 7.25 Hz, 2 H, NCH_2_), 2.92 (t, *J* = 7.25 Hz, 2 H, CH_2_).

#### 3.1.2. 1-(2-(Indolin-3-yl)ethyl)-3-(2,2,2-trichloro-1-hydroxyethyl)urea (Compound **4**)

A solution of compound **2** (0.5 g, 2.46 mmol) in THF (20 mL) was introduced in a vessel for MW experiments equipped with a heating plate and a magnetic bar. Chloral hydrate (compound **3**, 0.85 g, 5.14 mmol) was added to the solution and the mixture was heated at 90 °C for 2 h under MW irradiation (300 W).

After cooling and removal of the solvent in vacuo, the crude residue was subjected to FC on silica gel (eluent: ethyl acetate) to afford compound **4** (0.80 g, 93% yield) as ocker solid: m.p.: 138–140 °C; ^1^H-NMR (600 MHz, DMSO-d_6_, 25 °C) δ, ppm = 10.82 (s, 1 H, NH indole), 7.55 (d, *J* = 7.8 Hz, 1H), 7.35 (d, *J* = 7.3 Hz, 1H), 7.14 (d, *J* = 1.7 Hz, 1H), 7.07 (t, *J* = 7.8 Hz, 1H), 6.98 (t, *J* = 7.3 Hz, 1H), 6.78 (d, *J* = 9.6 Hz, 1H), 6.35 (t, *J* = 5.6 Hz, 1H), 5.63 (t, *J* = 9.6 Hz, 1H), 3.37 (m, 2H), 2.83 (m, 2H), (A very broad signal is overlapped to that at 7.35 ppm); ^13^C-NMR (150 MHz, CD_3_OD, 25 °C) δ, ppm = 156.3 (C), 136.3 (C), 127.2 (C), 122.8 (CH), 120.9 (CH), 118.4 (CH), 118.2 (CH), 111.7 (C), 111.3 (CH), 103.3 (C), 81.9 (CH), 39.8 (CH_2_), 25.8 (CH_2_); ATR-IR: (cm^−1^): 3401, 1658, 1558, 1074, 737; ESI-MS^+^ (*m*/*z*): 350 [M + H]^+^, 372 [M + Na]^+^; ESI-HRMS^+^ (*m*/*z*): for C_13_H_14_Cl_3_N_3_NaO_2_^+^ Calc. 372,00493, found 372.0049.

#### 3.1.3. 1-(2-(1*H*-Indol-3-yl)ethyl)-3-(2,2,2-trichloro-1-(3-(2-(3a,7a-dihydro-1*H*-indol-3-yl)ethyl)ureido)ethyl)urea (Compound **5**).

In a three necked round bottomed flask, partially immersed in an oil bath and equipped with a reflux condenser and a magnetic bar, chloral hydrate (**3**, 0.17 g, 1.03 mmol) was introduced under argon atmosphere. The system was heated at 100 °C until melting of compound **3**, then compound **2** (0.200 g, 0.984 mmol) and after 1 h anhydrous toluene (2 mL) was added and the reaction was stirred at 100 °C overnight and then allowed to cool to room temperature. After adding 10 mL of ethyl acetate the mixture was filtered under vacuum and the obtained solid was washed with methanol to obtain compound **5** as a grey solid. (132 mg, 0.246 mmol, 50%) m.p.: 208–210 °C; ^1^H-NMR (400 MHz, DMSO-d_6_, 25 °C) δ, ppm = 10.81 (s, 2 H, NH indole), 7.55 (d, *J* = 7.8 Hz, 2 H), 7.33 (d, *J* = 8.1 Hz, 2 H), 7.14 (d, *J* = 2.4 Hz, 2 H, CHNH), 7.06 (ddd, *J* = 8.1 Hz, *J* = 7.0 Hz, *J* = 1.2 Hz, 2 H), 6.97 (ddd, *J* = 7.8 Hz, *J* = 7.0 Hz, *J* = 1.0 Hz 2 H), 6.78 (d, *J* = 9.5 Hz, 2 H, NHCHCCl_3_), 6.33 (t, *J* = 9.5 Hz, 1 H, CHCCl_3_), 6.25 (t, *J* = 5.6 Hz, 2 H, NHCH_2_), 2.81 (td *J_1_* = 6.9 Hz, *J_2_* = 2.71 Hz, 4 H, CH_2_CH_2_N), (a signal is overlapped to that of water); ^13^C-NMR (100 MHz, DMSO-d_6_, 25 °C) δ, ppm = 155.9, 136.3, 127.2, 122.7 (CH), 120.9 (CH), 118.4 (CH), 118.2 (CH), 111.7, 111.3 (CH), 103.8, 67.6 (CH), 40.2 (CH_2_), 25.8 (CH_2_); ATR-IR: (cm^−1^): 3386, 3329, 3286, 1637, 1558, 740; ESI-MS^+^ (*m*/*z*): 535 [M + H]^+^ 557 [M + Na]^+^, 575 [M + K]^+^; ESI-HRMS^+^ (*m*/*z*): for C_24_H_25_Cl_3_N_6_NaO_2_^+^ Calc. 557.10023, found 557.1002.

#### 3.1.4. 2,2,2-Trichloro-1-(pyrimidin-2-ylamino)ethan-1-ol (Compound **7**)

A solution of 2-aminopyrimidine (compound **6**, 0.285 g, 3.0 mmol) and chloral hydrate (**3**, 496.5 mg, 3.0 mmol) in THF (20 mL) was introduced in a wessel for MW experiments equipped with a heating plate and a magnetic bar. The mixture was heated at 90 °C for 3 h under MW irradiation (300 W) then allowed to stand at room temperature. The solvent was removed in vacuo and the residue was subjected to bulb-to-bulb distillation (80 °C, 0.01 mmHg, 2 h). Pure compound **7** was recovered as white solid in the initial bulb (0.611 g, 2.52 mmol, 84% yield). m.p.:166.5–168.0 °C (Lit: [38] 167–169 °C); ^1^H-NMR (600 MHz, DMSO-d_6_, 25 °C) δ, ppm = 8.40 (d, *J* = 4.8 Hz, 2 H), 7.50 (m, 2 H, NH+OH), 6.79 (t, *J* = 4.8 Hz, 1 H), 6.20 (dd *J_1_* = 9.7 Hz, *J_2_* = 5.9 Hz, 1 H, CHCCl_3_); ^13^C-NMR (150 MHz, DMSO-d_6_, 25 °C) δ, ppm = 160.9, 158.1 (CH), 112.4 (CH), 103.1, 82.5 (CH); ATR-IR: (cm^-1^): 3300, 1576, 1074, 794; ESI-MS^+^ (*m*/*z*): 242 [M + H]^+^, 264 [M + Na]^+^; ESI-HRMS^+^ (*m*/*z*): for C_6_H_7_Cl_3_N_3_O^+^ Calc. 241.96547, found 241.9655.

#### 3.1.5. *N*-(1,2,2,2-tetrachloroethyl)pyrimidin-2-amine (Compound **8**)

In a flame-dried apparatus, equipped with a reflux condenser, dropping funnel and kept under inert atmosphere, a solution of compound **7** (241 mg, 1 mmol) in anhydrous THF (20 mL) was added. A solution of SOCl_2_ (73 μL, 1 mmol) in anhydrous THF (2 mL) was put in the dropping funnel and slowly added (dropwise in 20 min.). During and after the addition of SOCl_2_ nitrogen was bubbled into the solution in order to remove the gaseous by-products. The reaction course was monitored through ^1^H-NMR spectroscopy. When the conversion resulted quantitative, the solvent was removed and the white solid obtained was characterized and used without further purification. m.p.: 116.3–118.8 °C; ^1^H-NMR (300 MHz, DMSO-d_6_, 25 °C) δ, ppm = 8.45 (d, *J* = 4.6 Hz, 2 H), 7.74 (d, *J* = 9.5 Hz, 1 H, NH), 6.84 (t, *J* = 4.6 Hz, 1 H), 6.21 (d*, J* = 9.5 Hz, 1 H, CHCCl_3_); ^13^C-NMR (150 MHz, DMSO-d_6_, 25 °C) δ, ppm = 160.2, 158.0 (CH), 112.4 (CH), 102.9, 82.4 (CH); GC-MS (*m*/*z*): 189 (18, M-70), 154 (100, M-105), 119 (10, M-140), 79 (20); ESI-MS^+^ (*m*/*z*): 224 [M − Cl]^+^.

#### 3.1.6. 2,2,2-Trichloro-*N*-(2-(3a,7a-dihydro-1*H*-indol-3-yl)ethyl)-*N*′-(pyrimidin-2-yl)ethane-1,1-diamine (Compound **9**)

Tryptamine (compound **1**, 0.200 g, 1.25 mmol) in anhydrous THF (5 mL) was added to a solution of compound **8** (0.120 mg, 0.5 mmol) in anhydrous THF (5 mL). The reaction mixture was heated at 50 °C overnight. After removal of the solvent, the crude was subjected to FC on neutral alumina (changing gradient eluent from *n*-hexane/ethyl acetate 1/1 until pure ethyl acetate) and 44.2 mg (0.115 mmol, 23% yield) of pure compound **9**, as waxy brown solid was obtained. m.p.: 132.5–134.5 °C; ^1^H-NMR (400 MHz, DMSO-d_6_, 25 °C) δ, ppm = 10.76 (br. s, 1 H, NH indole), 8.37 (d, *J* =4.7 Hz, 2 H), 7.56 (d, *J* = 9.1 Hz, 1 H), 7.39 (d, *J* = 7.8 Hz, 1 H), 7.30 (dt, *J_1_* = 8.0, *J_2_* = 0.8 Hz, 1 H), 7.11 (d, *J* = 2.3 Hz, 1 H), 7.03 (ddd, *J* = 8.0 Hz, *J* = 7.0 Hz, *J* = 1.2 Hz, 1 H), 6.91 (ddd, *J* = 7.8 Hz, *J* = 7.0 Hz, *J* = 1.0 Hz, 1 H), 6.73 (t, *J* = 4.7 Hz, 1 H), 5.70 (dd, *J_1_* = 10.8, *J_2_* = 9.1 Hz, 1 H, CHCCl_3_), 3.04–2.76 (m, 4 H); ^13^C-NMR (100 MHz, DMSO-d_6_, 25 °C) δ, ppm = 162.0, 158.1 (CH), 157.9, 136.1, 127.1 (CH), 122.6 (CH), 120.8 (CH), 118.1 (CH), 111.9, 111.8 (CH), 111.3 (CH), 103.8, 76.3 (CH), 47.3 (CH_2_), 25.5 (CH_2_); ATR-IR: (cm^−1^): 3411, 3221, 1584, 1444, 1415, 798, 784, 740; ESI-MS^+^ (*m*/*z*): 384 [M + H]^+^, 406 [M+Na]^+^, 424 [M + K]^+^; ESI-HRMS^+^ (*m*/*z*): for C_16_H_16_Cl_3_N_5_Na^+^ Calc. 406.03690, found 406.0369.

#### 3.1.7. Methyl 9-((2-(1*H*-indol-3-yl)ethyl)amino)-9-oxononanoate (Compound **13**)

Tryptamine (compound **1**, 320.5 mg, 2 mmol) was added to a magnetically stirred solution of crude freshly prepared compound **12** (1 mmol) [17,18]. After 3 h at room temperature, ^1^H-NMR analysis of a sample revealed disappearance of the signals belonging to **12**. After removal of the solvent in vacuo and purification through FC (eluent: DCM/MeOH 9.5/0.5) 199.5 mg (0.58 mmol, 58%) of pure **13** was obtained as a light brown solid: m.p.:56.5–58.0 °C; ^1^H-NMR (300 MHz, CDCl_3_, 25 °C) δ, ppm = 8.60 (s, 1 H, NH), 7.59 (d, *J* = 7.8 Hz, 1 H), 7.37 (d, *J* = 8.1 Hz, 1 H), 7.19 (t, *J* = 7.0 Hz, 1 H), 7.11 (t, *J* = 7.2 Hz, 1 H), 7.00 (s, 1 H), 5.65 (br.s, 1 H, NH), 3.67 (s, 3 H), 3.59 (q, *J* = 6.4 Hz, 2 H, CH_2_CH_2_NH), 2.97 (t, *J* = 6.4 Hz, 2 H), 2.29 (t, *J* = 7.3 Hz, 2 H, CH_2_COOCH_3_), 2.08 (t, *J* = 7.3 Hz, 2 H), 1.65–1.50 (m, 4 H), 1.26 (br.s, 6 H); ^13^C-NMR (100 MHz, CDCl_3_, 25 °C) δ, ppm = 174.4, 173.5, 136.4, 127.3, 122.1 (CH), 122.0 (CH), 119.3 (CH), 118.5 (CH), 112.6, 111.3 (CH), 51.5 (CH_3_), 39.7 (CH_2_), 36.5 (CH_2_), 34.0 (CH_2_), 28.9 (CH_2_, 2 signals overlapped), 28.8 (CH_2_), 25.6 (CH_2_), 25.2 (CH_2_), 24.7 (CH_2_); ATR-IR: (cm^−1^): 3390, 3272, 1730, 1633, 737; ESI-MS^+^ (*m*/*z*): 345 [M + H]^+^, 367 [M + Na]^+^, 383 [M + K]^+^; ESI-HRMS^+^ (*m*/*z*): for C_20_H_28_N_2_NaO_3_^+^ Calc. 367.19976, found 367.1998.

#### 3.1.8. 9-((2-(1*H*-Indol-3-yl)ethyl)amino)-9-oxononanoic Acid (Compound **14**)

LiOH·H_2_O (25 mg, 0.6 mmol) was added to a solution of compound **13** (0.172 g, 0.5 mmol) in 25 mL of a 20% *v*/*v* solution of THF in H_2_O and the mixture was stirred at room temperature for 3 h. After acidification with 10% aq. HCl, EtOAc and H_2_O were added to the mixture. The extracted organic layer was dried over anhydrous MgSO_4_ and concentrated and pure compound **14** was recovered in 86% yield (142 mg, 0.41 mmol) as pale grey solid: m.p.: 88.0–91.4 °C; ^1^H-NMR (300 MHz, CDCl_3_, 25 °C) δ, ppm = 8.42 (br.s, 1 H, NH), 7.58 (d, *J* = 7.8 Hz, 1 H), 7.37 (d, *J* = 7.8 Hz, 1 H), 7.19 (dt, *J_1_* = 7.8 Hz, *J_2_* = 1.2 Hz 1 H), 7.11 (dt, *J_1_* = 7.8 Hz, *J_2_* = 1.2 Hz 1 H), 7.01 (d, *J* = 1.9 Hz, 1 H), 5.63 (br.s, 1 H, NH), 3.60 (q, *J* = 6.2 Hz, 2 H), 2.96 (t, *J* = 7.0 Hz, 2 H), 2.38–2.28 (m, 4 H), 2.09 (t, *J* = 6.2, 2H) 1.69–1.48 (m, 6 H), 1.39–1.29 (br.s, 2 H); ^13^C NMR (75 MHz, CDCl_3_, 25 °C) δ, ppm = 179.0, 173.5, 173.4, 127.3, 122.2 (CH), 122.1 (CH), 119.4 (CH), 118.6 (CH), 112.7, 111.3 (CH), 39.6 (CH_2_), 36.7 (CH_2_), 33.9 (CH_2_), 30.3 (CH_2_), 28.8 (CH_2_), 28.7 (CH_2_), 25.5 (CH_2_), 25.2 (CH_2_), 24.6 (CH_2_); ^1^H-NMR (600 MHz, CD_3_CN, 25 °C) δ, ppm = 9.17 (br.s, 1 H, NH), 7.59 (d, *J* = 8.0 Hz, 1 H), 7.40 (d, *J* = 8.2 Hz, 1 H), 7.14 (t, *J* = 8.2 Hz, 1 H), 7.09 (d, *J* = 1.6 Hz, 1 H), 7.06 (t, *J* = 8.0 Hz, 1 H), 6.53 (br.s, 1 H, NH), 3.46 (q, *J* = 7.0 Hz, 2 H), 2.91 (t, *J* = 7.2 Hz, 2 H), 2.27 (t, *J* = 7.6, 2H), 2.09 (t, *J* = 7.7 hZ, 2H), 1.60–1.48 (m, 4 H) 1.34–1.20 (m, 6 H); ^13^C-NMR (150 MHz, CD_3_CN, 25 °C) δ, ppm = 175.4, 174.1, 137.5, 128.5, 123.5 (CH), 122.3 (CH), 119.7 (CH), 119.4 (CH), 113.4, 112.2 (CH), 40.5 (CH_2_), 36.8 (CH_2_), 34.2 (CH_2_), 29.60 (CH_2_), 29.58 (CH_2_), 29.54 (CH_2_), 26.4 (CH_2_), 26.0 (CH_2_), 25.5 (CH_2_); ATR-IR: (cm^−1^): 3388, 3269, 1710, 1631; ESI-MS^+^ (*m*/*z*): 331 [M + H]^+^, 353 [M + Na]^+^, 369 [M + K]^+^; ESI-HRMS^+^ (*m*/*z*): for C_19_H_26_N_2_NaO_3_^+^ Calc. 353.18411, found 353.1841.

### 3.2. Cell Culture and Treatments

#### 3.2.1. Cell Culture

The squamous carcinoma (A431), human colon cancer (HT29), human bone osteosarcoma (U2OS), a human T-cell acute lymphoblastic leukemia (Jurkat 6) and acute myeloid leukemia (MV-4-11) cell lines were purchased from American Type Culture Collection (ATCC, Manassas, VA). Two additional human cell lines, a B-cell acute lymphoblastic leukemia (REH) and an acute myeloid leukemia (KG-1) model were obtained from Leibniz-Institut DSMZ-Deutsche Sammlung von Mikroorganismen und Zellkulturen GmbH (Germany) and the human ovarian cancer cell line (IGROV1) has been kindly provided by Istituto Nazionale Tumori (IRCCS, Milan, Italy). Cells were cultured in RPMI 1640 medium (Labtek Eurobio, Milan, Italy), supplemented with 10% heat-inactivated fetal bovine serum (Thermo Fisher Scientific, Waltham, MA, USA) or fetal calf serum (Euroclone, Milan, Italy), 100 U/mL penicillin, 100 μg/mL streptomycin (GE Healthcare, Chicago, IL, USA) and 2mM l-glutamine (Sigma-Aldrich, Milan, Italy), at 37 °C and 5% CO_2_ atmosphere. The compounds were dissolved in DMSO (Sigma-Aldrich, Milan, Italy) in a stock solution. 

#### 3.2.2. Cell Viability Assays 

To evaluate the compounds activity, the cells were treated for 24 h with vehicle (DMSO, as control) or with the test samples at concentrations between 0.01 µM and 250 µM. Cell growth was assessed by the luminescence-based RealTime-Glo MT Cell Viability Assay (Promega, Madison, WI, USA) or the colorimetric 3-(4,5-dimethylthiazolyl-2)-2,5-diphenyltetrazolium bromide assay (MTT, Sigma-Aldrich, Milan, Italy). RealTime-Glo MT Cell Viability Assay was performed according to manufacturer instructions and luminescence was quantified by using GloMax 96 Microplate Luminometer (Promega, Madison, WI, USA). As for MTT assays, the culture medium was removed and cells incubated with 0.1 mL of MTT dissolved in PBS at the concentration of 0.2 mg/mL following Micheletti et.al. [16]. The absorbance at 570 nm was measured using a multiwell plate reader (Tecan, Männedorf, Switzerland). The IC_50_ was determined from the dose-response curve by using Graph Pad software v.6.0.

## 4. Conclusions

We synthesized five novel derivatives characterized by the tryptamine nucleus as the common structural motif. This heterocycle was bound to an azelayl chain through an amide bond or to a 1,1,1-trichloroethyl group, in turn connected to another heterocyclic scaffold. In particular, ureido derivatives **4** and **5** bearing one and two tryptamine groups, respectively, were prepared by reaction of 1-(2-(1*H*-indol-3-yl)ethyl)urea with chloral hydrate. Reaction of 2-aminopyrimidine with chloral hydrate gave compound **7** that, after treatment with thionyl chloride and subsequent nucleophilic attack by tryptamine, produced compound **9**. The combination of tryptamine and azelaoyl scaffolds under Schotten-Baumann like conditions produced structural hybrids compounds **13** and **14**, a methyl ester and its free acid form, respectively, whose structures recall those of compounds behaving as HDAC inhibitors. 

Additionally, the biological activity of these compounds was investigated on a panel of solid and hematological cancer cell lines and the data reported suggest that their cellular toxicity could be related to different mechanisms involved in different biological targets. 

Compound **9** shows a marked cytotoxicity on leukemic cell lines tested, with IC_50_ values comprised from 28.5 µM for MV-4-11 to 0.570 µM for Jurkat 6; was inactive only in KG-1. In all solid tumor lines tested it is inactive, except for the U2OS cell line where it induces cytotoxic effect at high concentrations (IC_50_ = 235 µM). 

The binuclear compound **5** loses activity in leukemic and solid tumor cell lines, acting only in HT29 at high concentrations (IC_50_ = 212 µM). 

Compound **4** loses activity in all leukemic lines, while in solid tumors shows a cytotoxic effect only in HT29 (IC_50_ = 0.0115 µM) and in U2OS (IC_50_ = 22.54 µM). 

These data confirm that both the triptaminyl and the pyrimidinyl moieties connected through the trichloroehtyl group (compound **9**) are relevant to the compound effectiveness against acute leukemia models, while showing a modest and selective activity in solid cancers. 

Interestingly, keeping constant the presence of the tryptaminic scaffold and binding it to the azelayl moiety, the compounds maintain a biological activity. 

Compound **13** is active against KG-1 (IC_50_ = 32.44 µM), MV-4-11 (IC_50_ = 102.50 µM), REH (IC_50_ = 29.32 µM) and a selective effect only against HT29 cells (IC_50_ = 0.006 µM), among solid tumor models.

Compound **14** loses activity on all leukemic lines, while showing a marked cytotoxicity on all solid tumor lines tested, with IC_50_ values of 0.0072 µM for A431, 0.096 µM for HT29, 0.0015 for IGROV1 and 0.469 for U2OS. 

Studies addressing the clarification and/or identification of additional biological targets as well as the deepen investigation of structure–activity relationships are currently ongoing in our laboratories.

## Figures and Tables

**Table 1:** IC_50_ (μM) values of the new compounds in the analyzed tumor cell lines ^a^.

	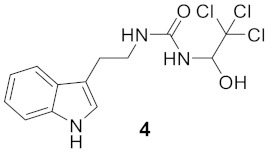	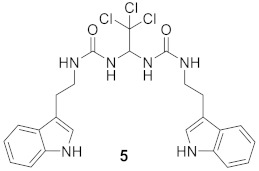	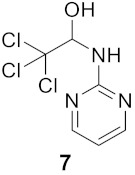	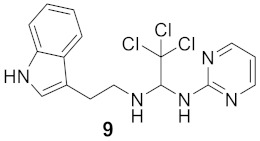	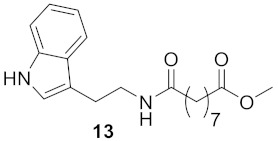	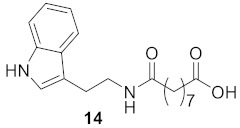
A431	n.a	n.a	n.a	n.a	>250	0.0072
HT29	0.0115	212	n.a	n.a	0.006	0.096
IGROV1	>250	n.a	n.a	n.a	>250	0.0015
U2OS	22.54	n.a	0.383	235	222	0.469
KG-1	n.a	n.a	n.a	n.a	32.44	n.a
MV-4-11	n.a	n.a	n.a	28.95	102.50	n.a
REH	n.a	n.a	n.a	65.32	29.32	n.a
Jurkat 6	n.a	n.a	61.12	0.570	n.a	n.a

^a^ n.a—not active.

## Data Availability

Not applicable.

## Supplementary Materials

The following are available online, Figures S1–S31: ^1^H-, ^13^C-NMR and ESI-Ms spectra.

## Sample Availability

Samples of compounds **7** and **13** are available from the authors.
