# Supplementary material for: Synthesis of Novel Tryptamine Derivatives and Their Biological Activity as Antitumor Agents
Source: Molecules. 2021 Jan 28;26(3):683. doi: 10.3390/molecules26030683 (PMC7865355; doi:10.3390/molecules26030683)
Supplement: Supplementary file 1 [file molecules-26-00683-s001.pdf]

Supplementary material

Synthesis of novel tryptamine derivatives and their biological activity as antitumor agents

Giorgia Simonetti [1], Carla Boga [2]*, Joseph Durante [3], Gabriele Micheletti [2], Dario Telese [2], Paolo Caruana [2], Andrea Ghelli Luserna di Rorà [1], Fabio Mantellini [3], Samantha Bruno [4], Giovanni Martinelli [1], and Natalia Calonghi [5]*

1. Biosciences Laboratory, IRCCS Istituto Romagnolo per lo Studio dei Tumori "Dino Amadori" - IRST S.r.l., 47014, Meldola (FC), Italy; giorgia.simonetti@irst.emr.it (G.S.); andrea.ghellilusernadirorà@irst.emr.it (A.G.); giovanni.martinelli@irst.emr.it (G.M.1)
2. Department of Industrial Chemistry "Toso Montanari", Alma Mater Studiorum – Università di Bologna, Viale del Risorgimento 4, 40136 Bologna, Italy; paolo.caruana@gidi.it (P.C.); gabriele.micheletti3@unibo.it (G.M.2); dario.telese2@unibo.it (D.T.)
3. Department of Biomolecular Sciences, University of Urbino "Carlo Bo",Via I Maggetti 24, 61029 Urbino (PU), Italy; fabio.mantellini@uniurb.it (F.M.); jdurante6@gmail.com (J.D.)
4. Department of Experimental, Diagnostic and Specialty Medicine, University of Bologna and Institute of Hematology and Medical Oncology "L. and A. Seràgnoli", 40138, Bologna, Italy;
samantha.bruno2@unibo.it (S.B.).
5. Department of Pharmacy and Biotechnology, University of Bologna, 40121 Bologna, Italy; natalia.calonghi@unibo.it (N.C.)

***** Correspondence: carla.boga@unibo.it (C.B.); natalia.calonghi@unibo.it (N.C.);
Tel.: +39-051-209-3616 (C.B.); +39-051-209-1231 (N.C.)

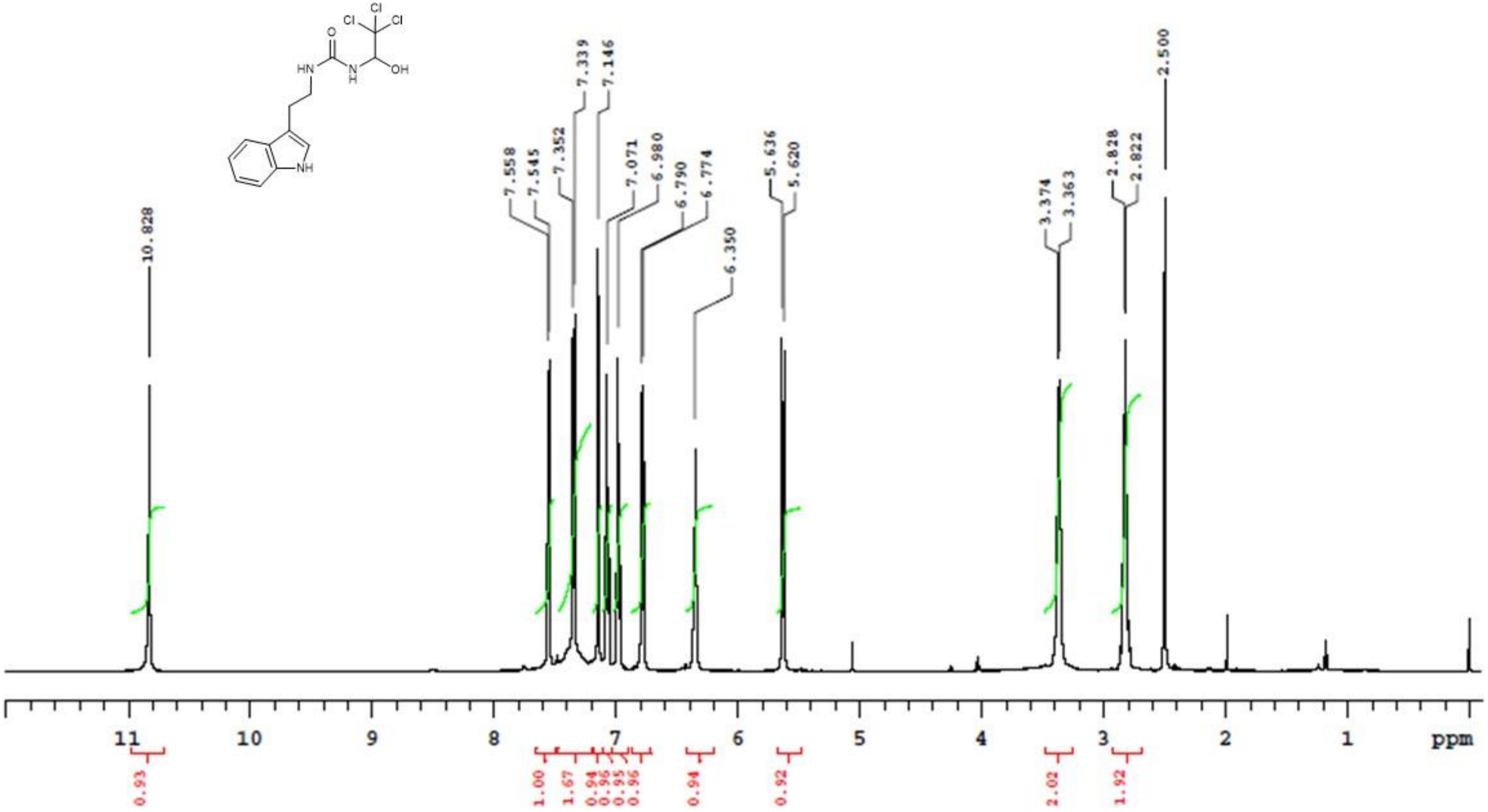

Figure S1. ^{1}H NMR spectrum of compound **4** in DMSO-d_6 (600 MHz, 25 °C)

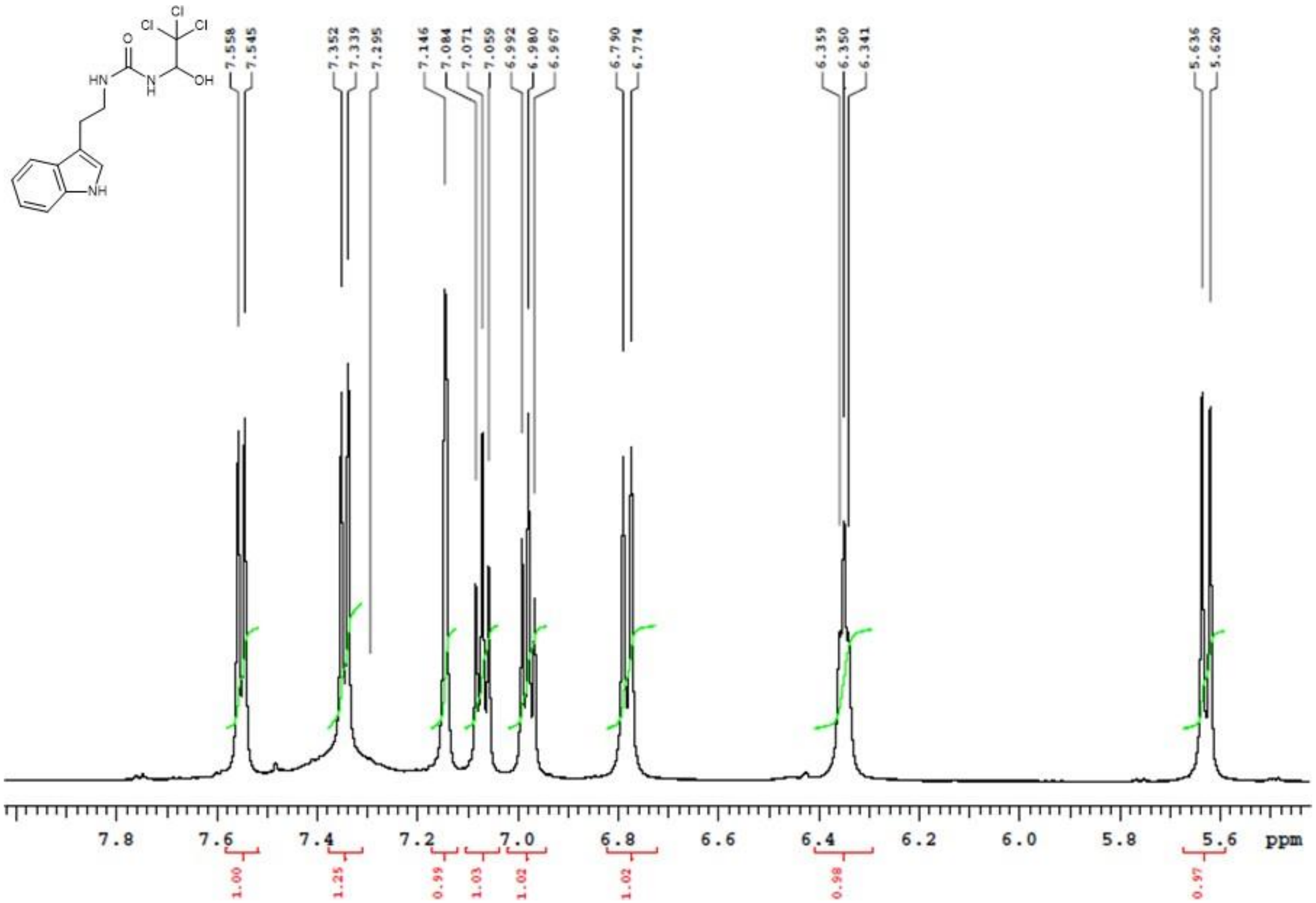

Figure S2. Expanded view of aromatic region of ^{1}H NMR spectrum of compound **4** in DMSO-d_6 (600 MHz, 25 °C)

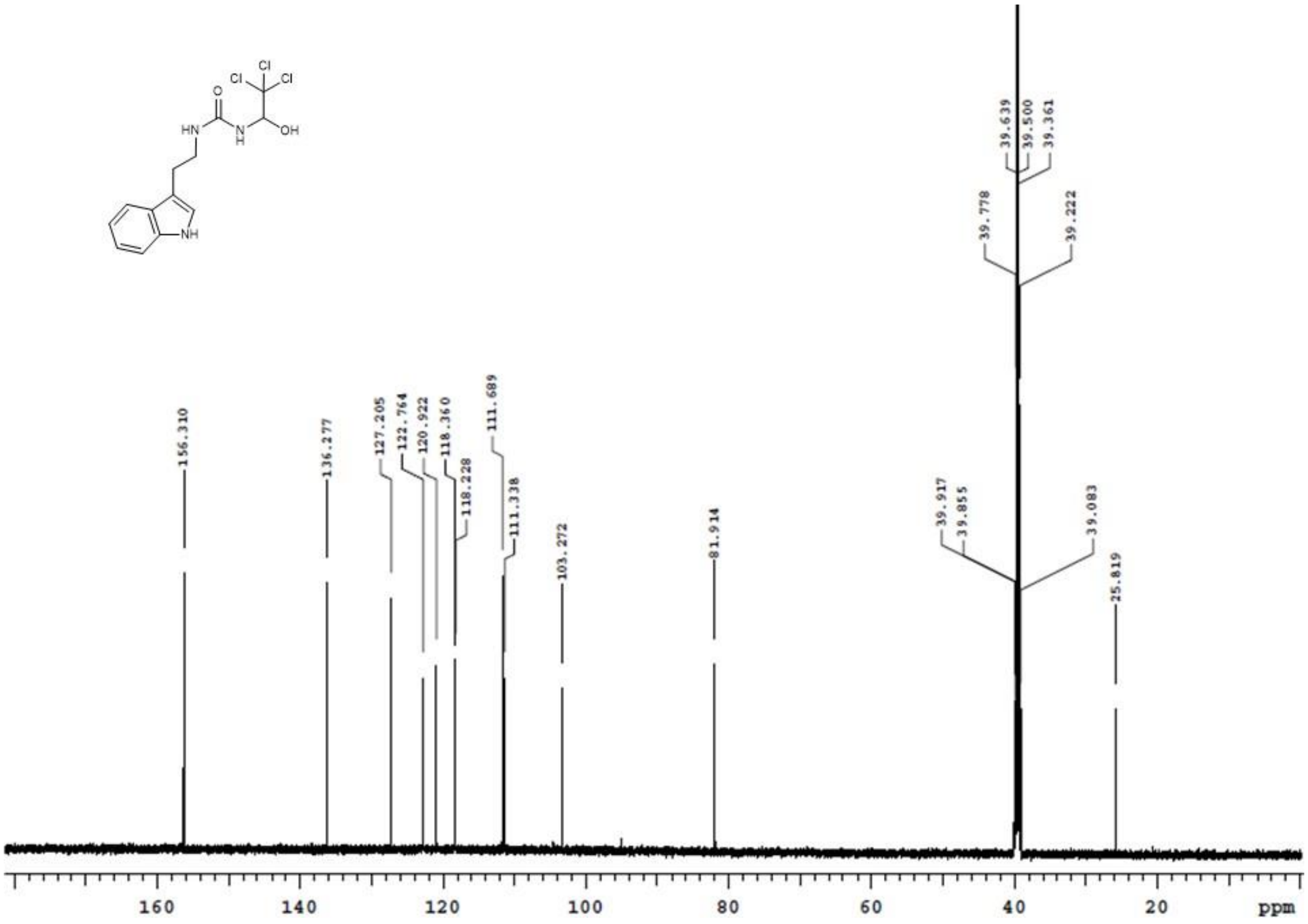

Figure S3. ^{13}C NMR spectrum of compound **4** in DMSO-d_6 (150 MHz, 25 °C)

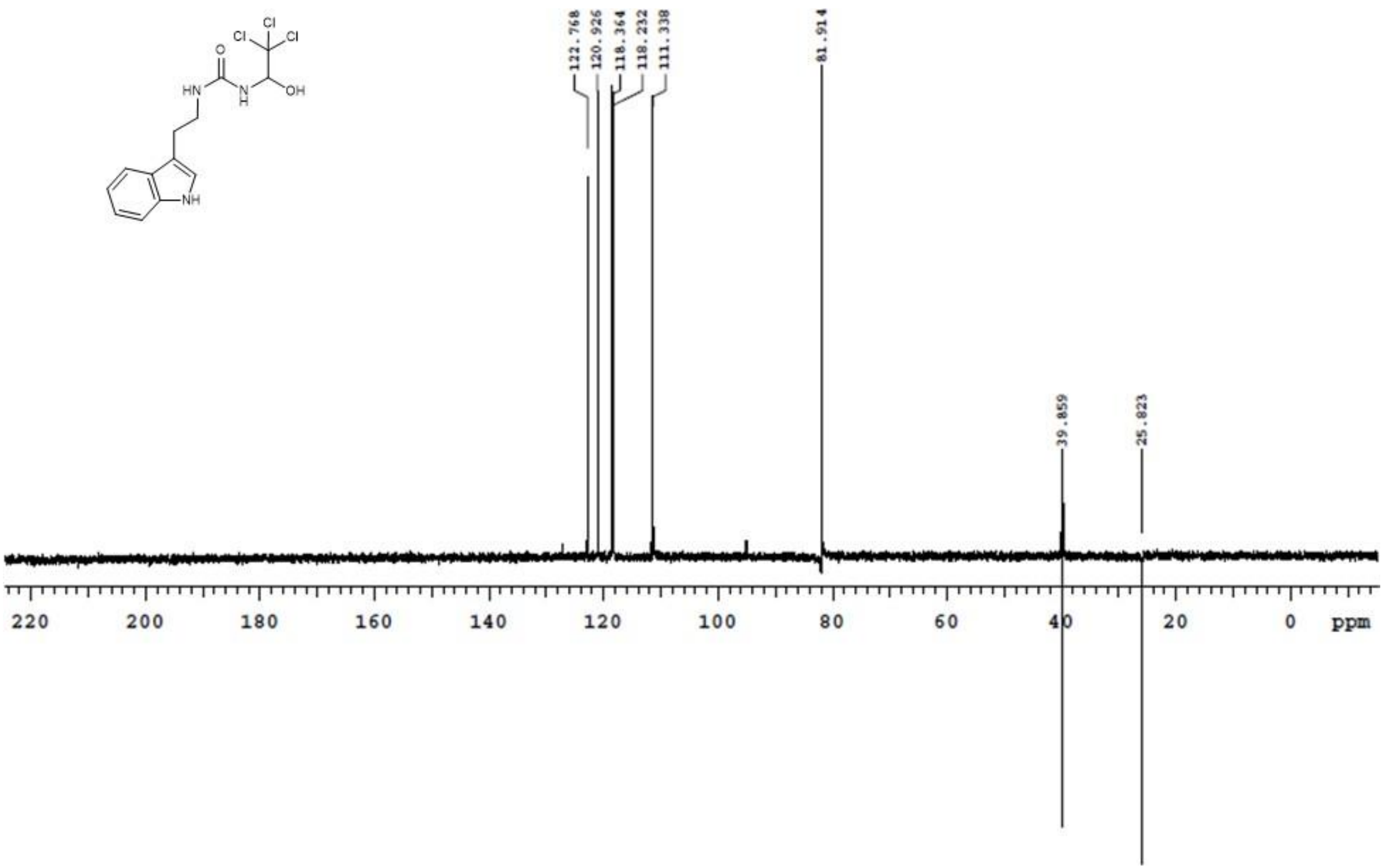

Figure S4. DEPT spectrum of compound **4** in DMSO-d$_6$ (150 MHz, 25 °C)

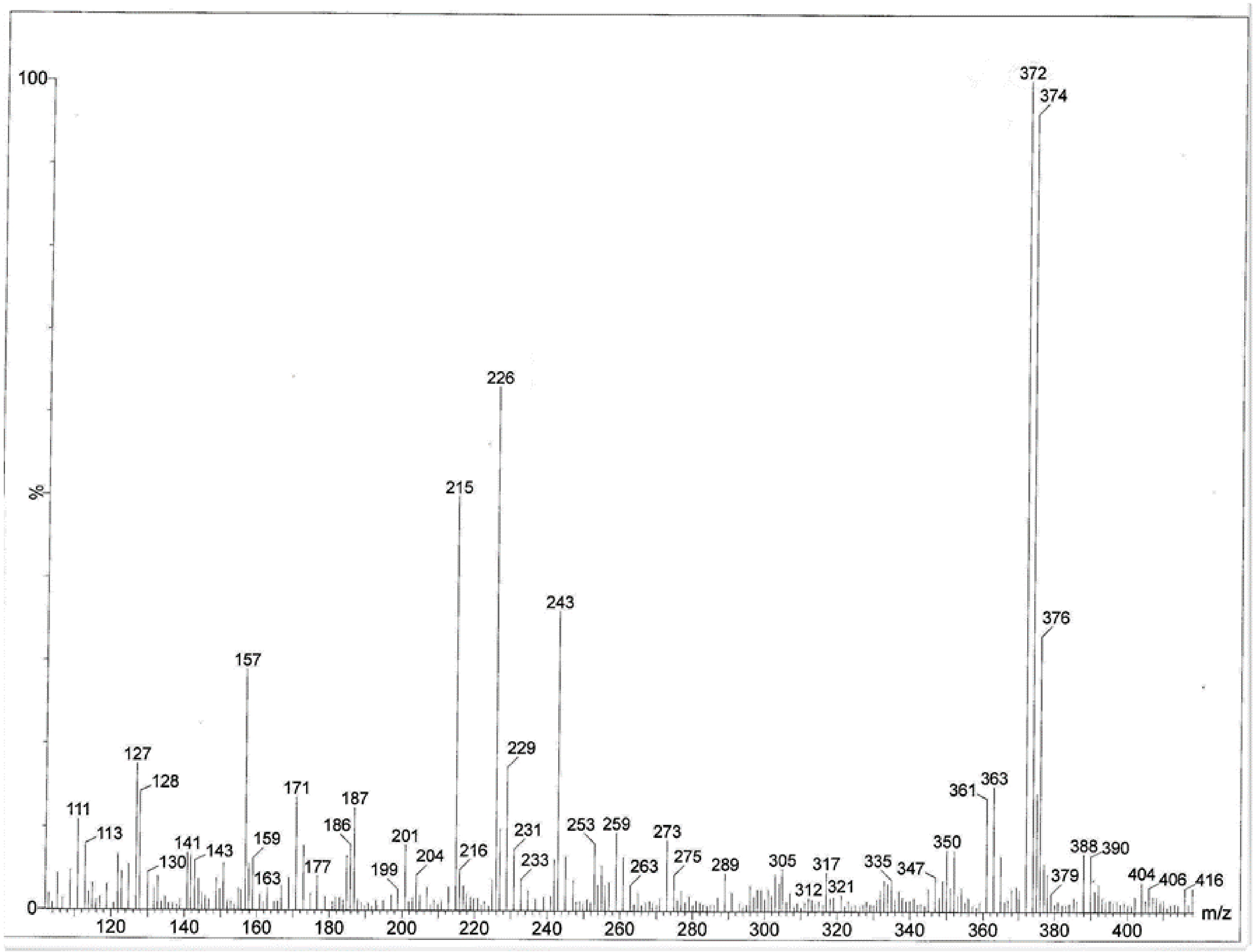

Figure S5. ESI-MS+ spectrum of compound **4**.

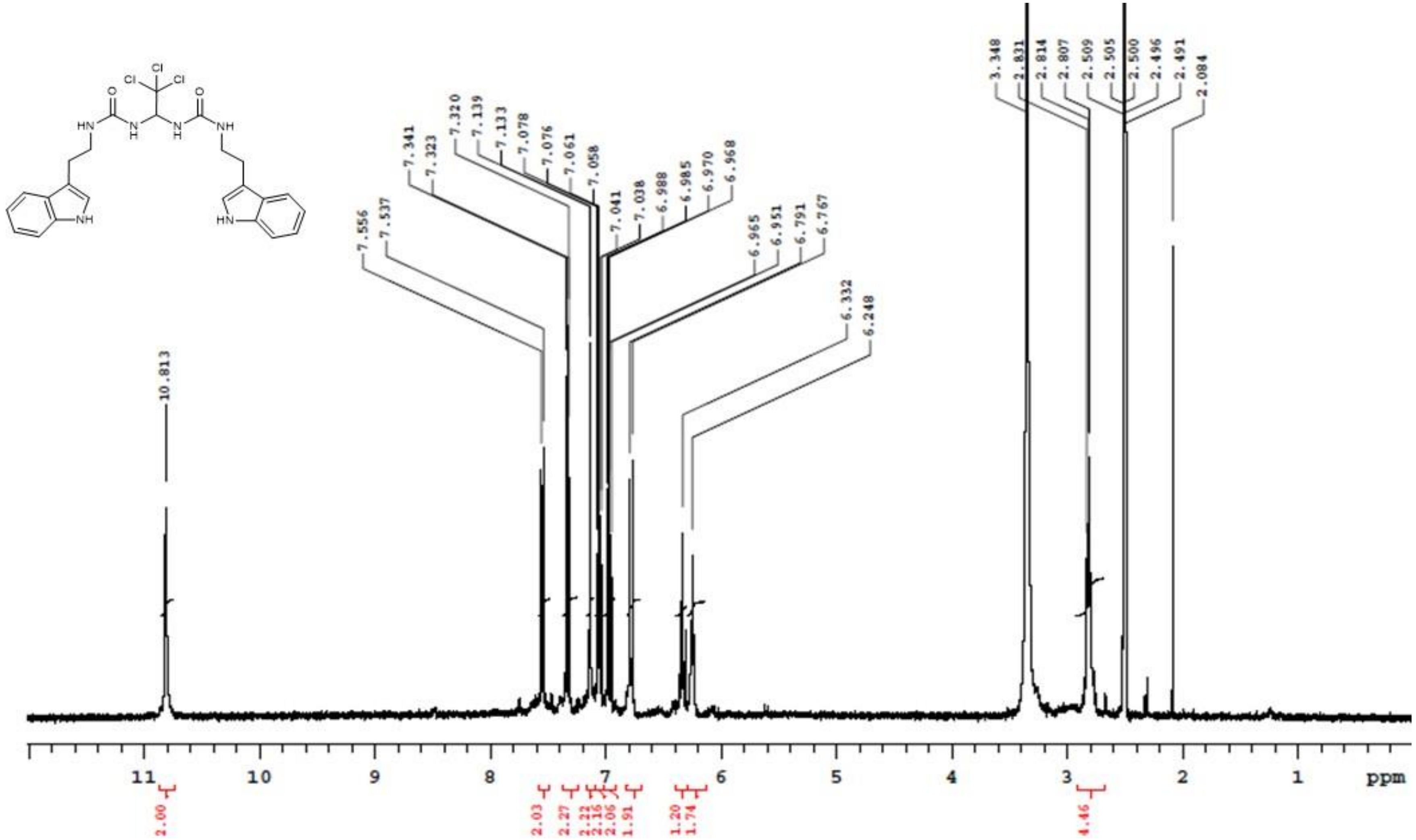

Figure S6. ^{1}H NMR spectrum of compound **5** in DMSO-d_6 (400 MHz, 25 °C)

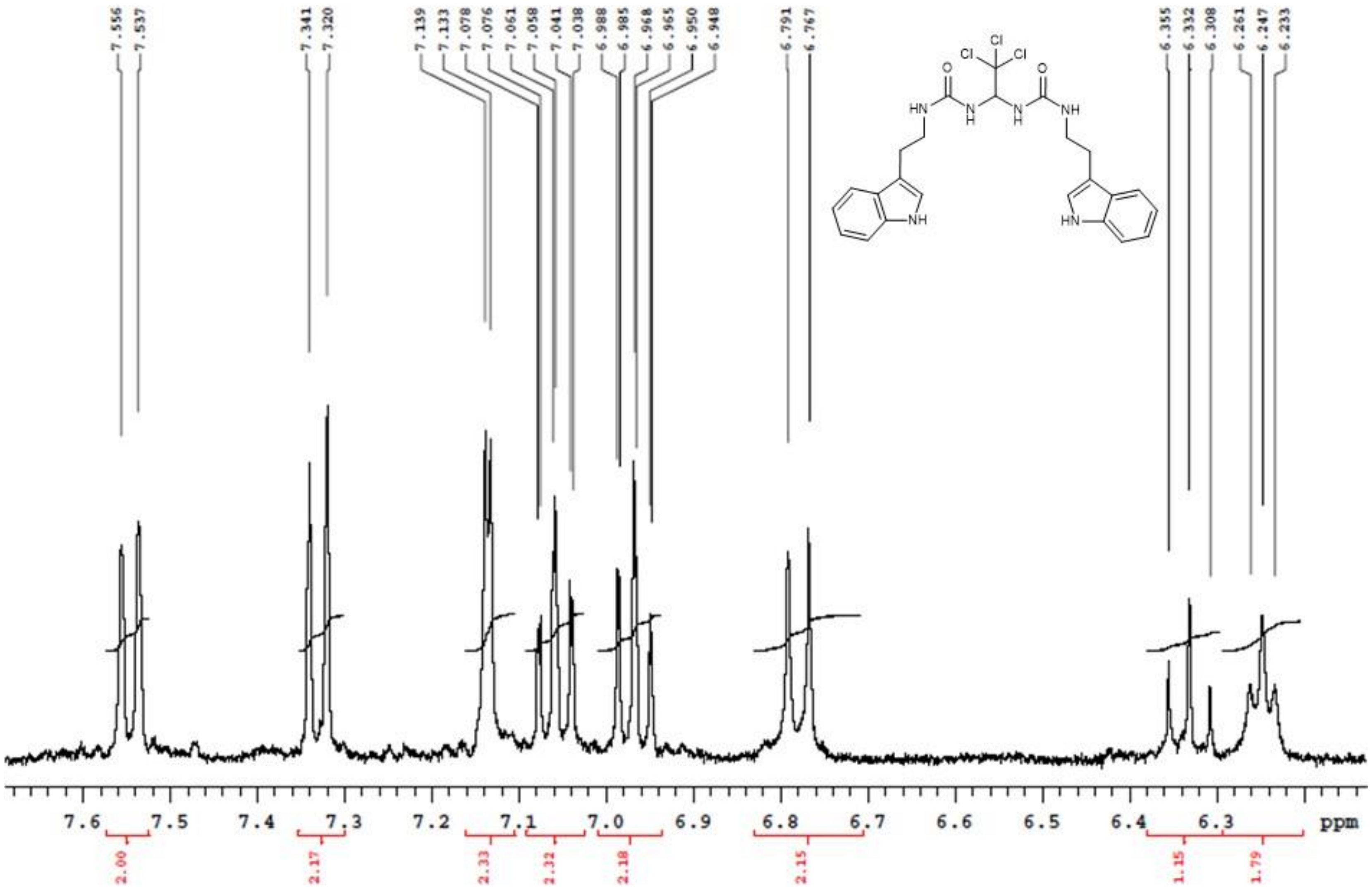

Figure S7. Expanded view of aromatic region of ^{1}H NMR spectrum of compound **5** in DMSO-d_6 (400 MHz, 25 °C)

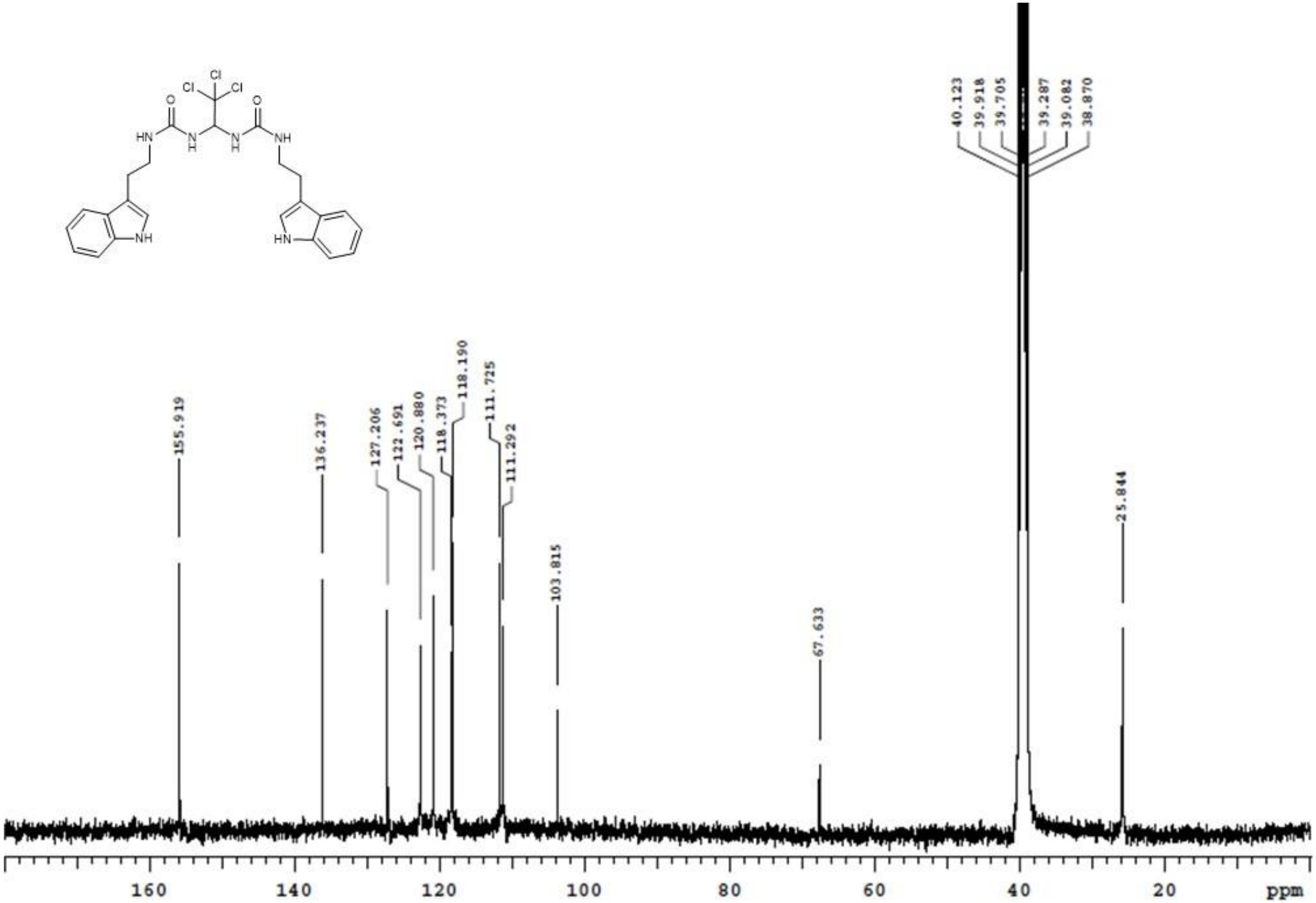

Figure S8. ^{13}C NMR spectrum of compound **5** in DMSO-d_6 (100 MHz, 25 °C)

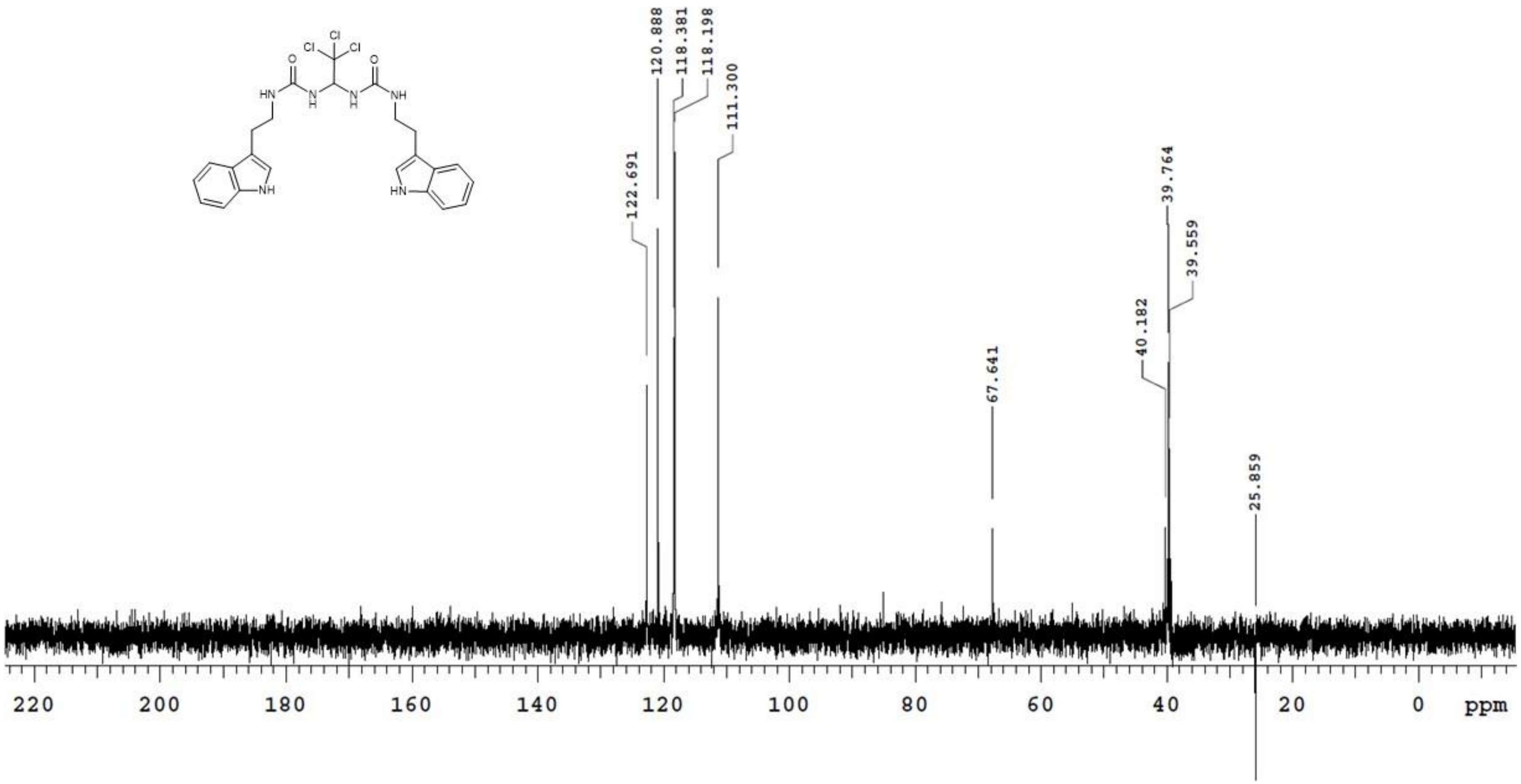

Figure S9. DEPT spectrum of compound **5** in DMSO-d_6 (100 MHz, 25 °C)

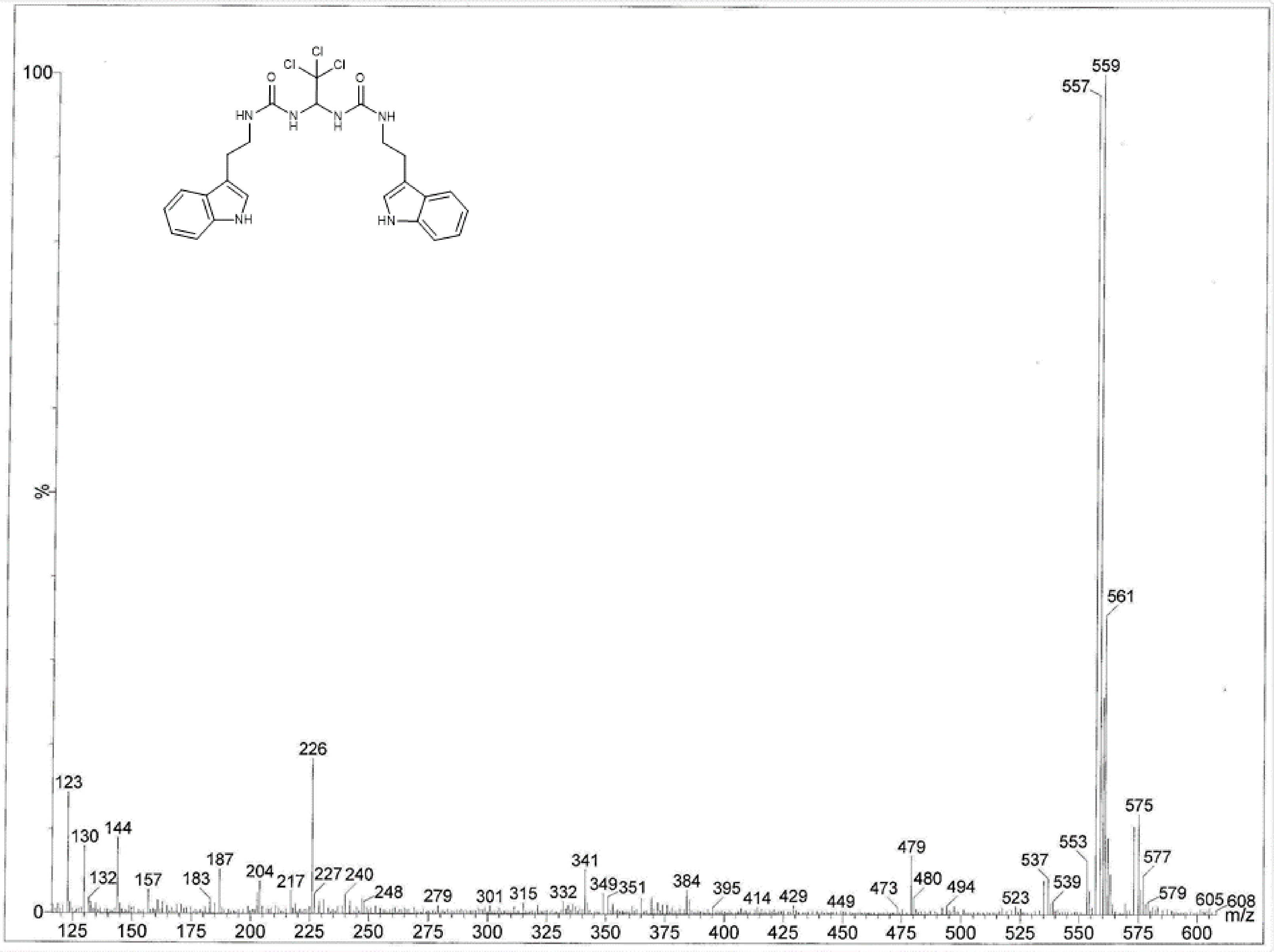

Figure S10. ESI-MS^+ spectrum of compound **5**.

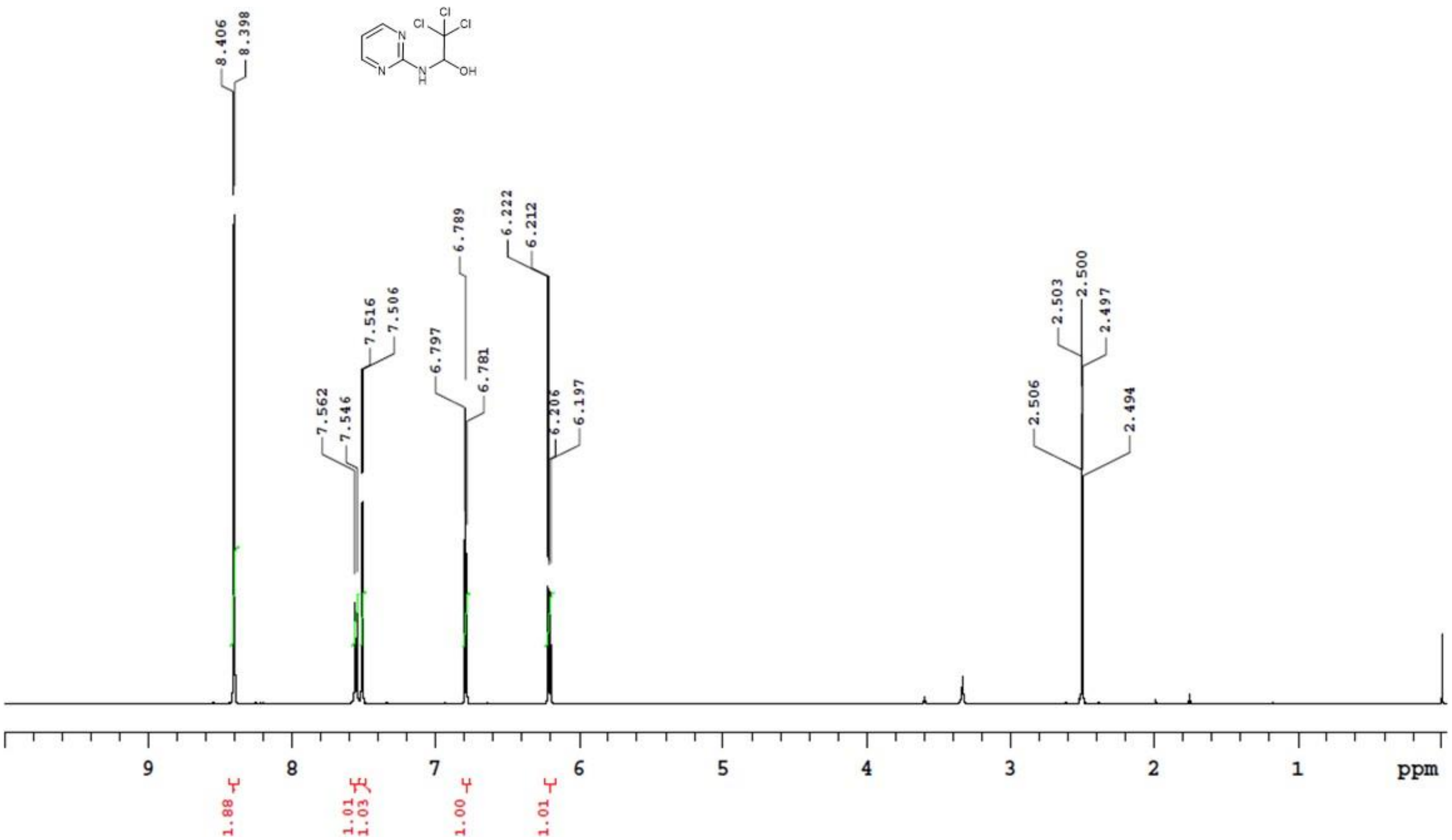

Figure S11. ^{1}H NMR spectrum of compound **7** in DMSO-d_6 (600 MHz, 25 °C).

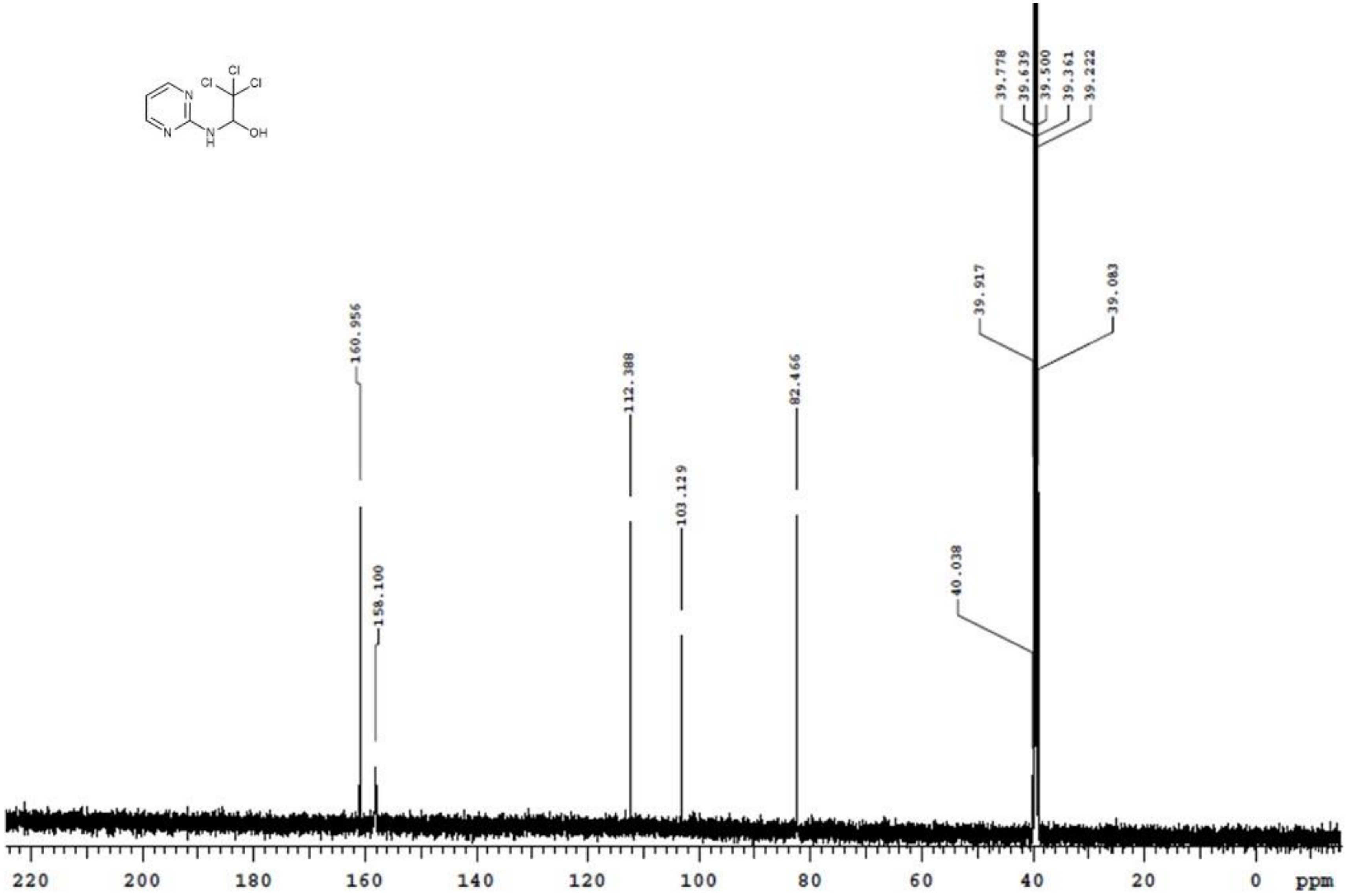

Figure S12. ^{13}C NMR spectrum of compound **7** in DMSO-d_6 (150 MHz, 25 °C)

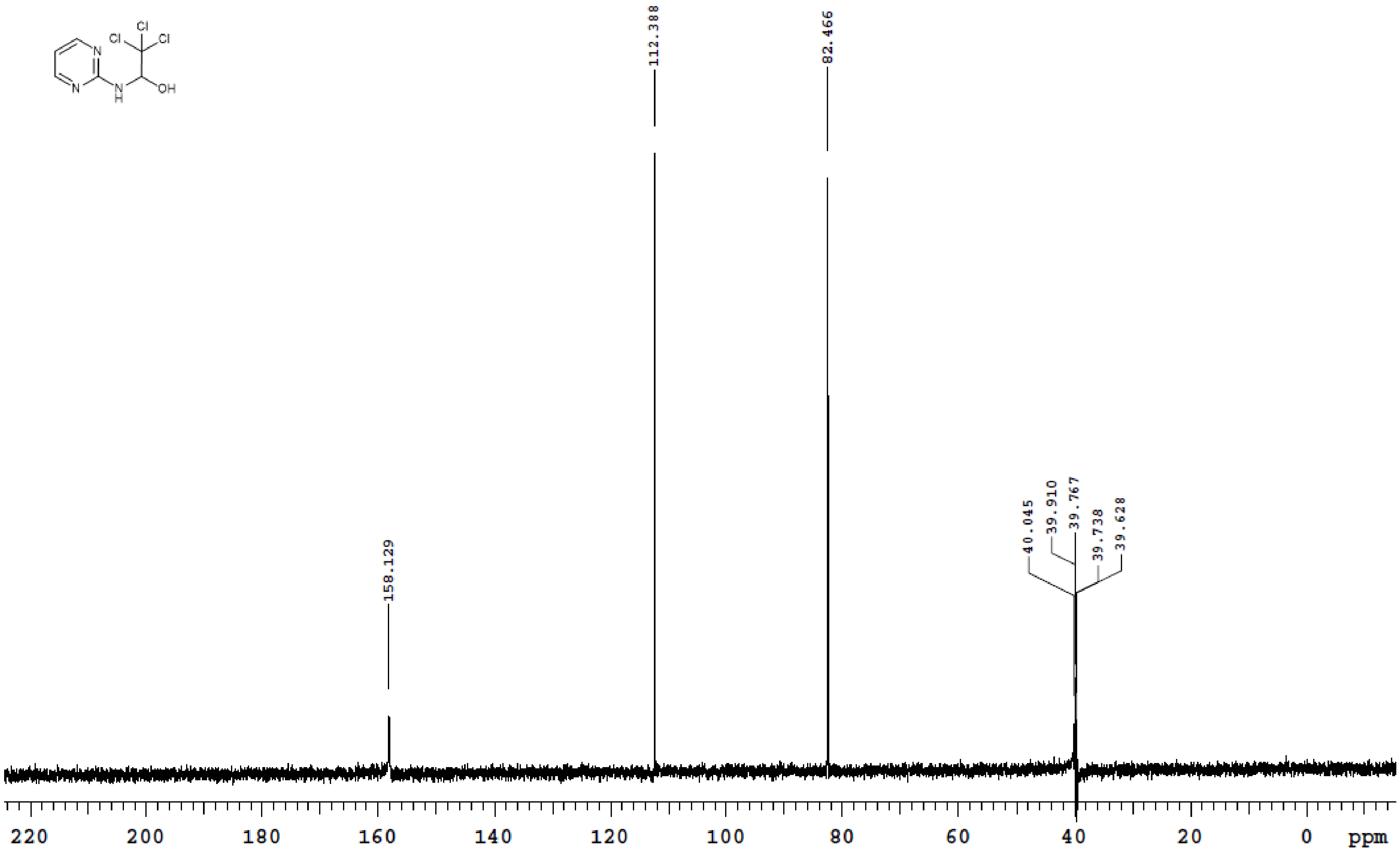

Figure S13. DEPT spectrum of compound **7** in DMSO-d_6 (150 MHz, 25 °C)

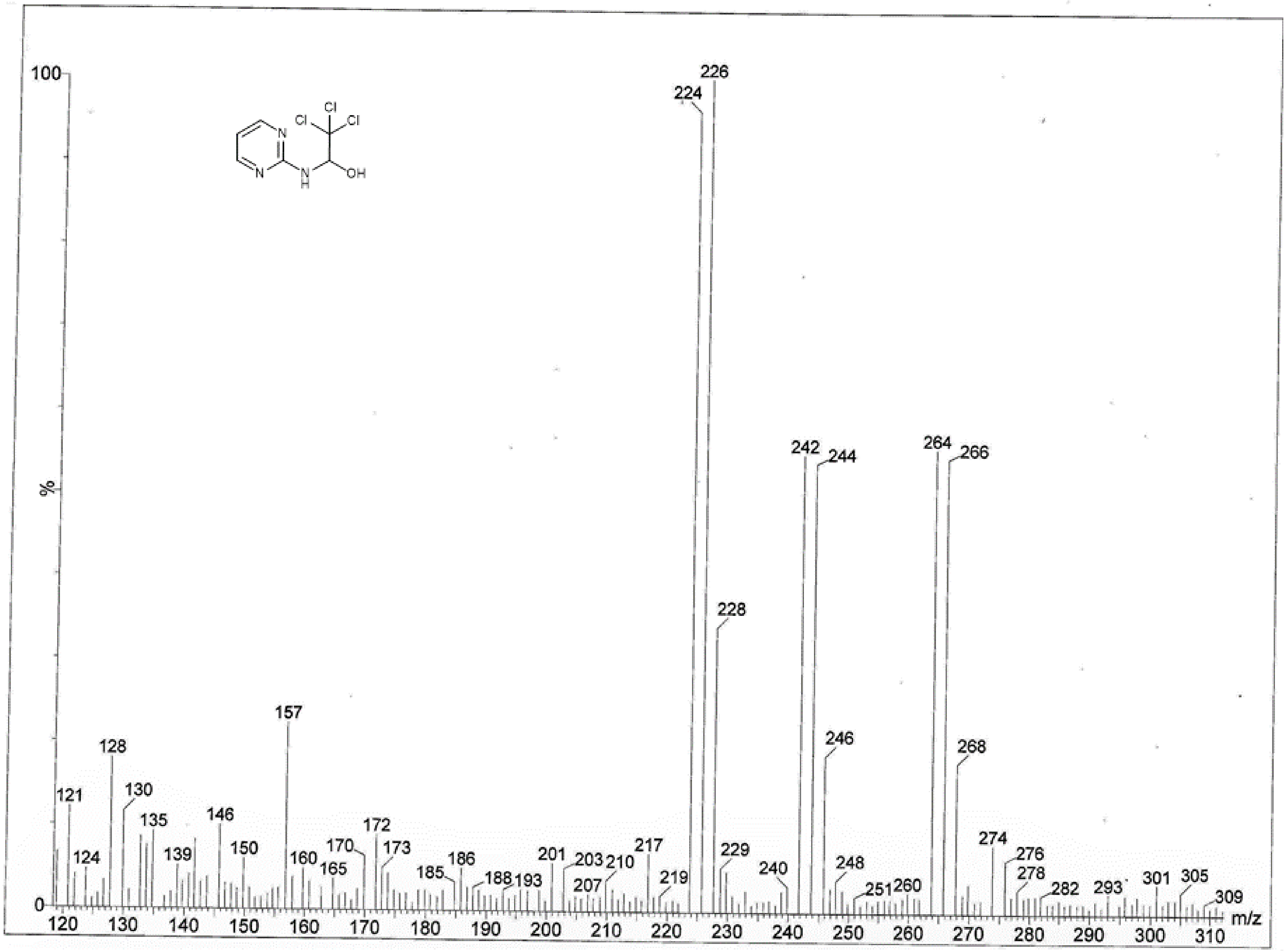

Figure S14. ESI-MS$^+$ spectrum of compound **7**.

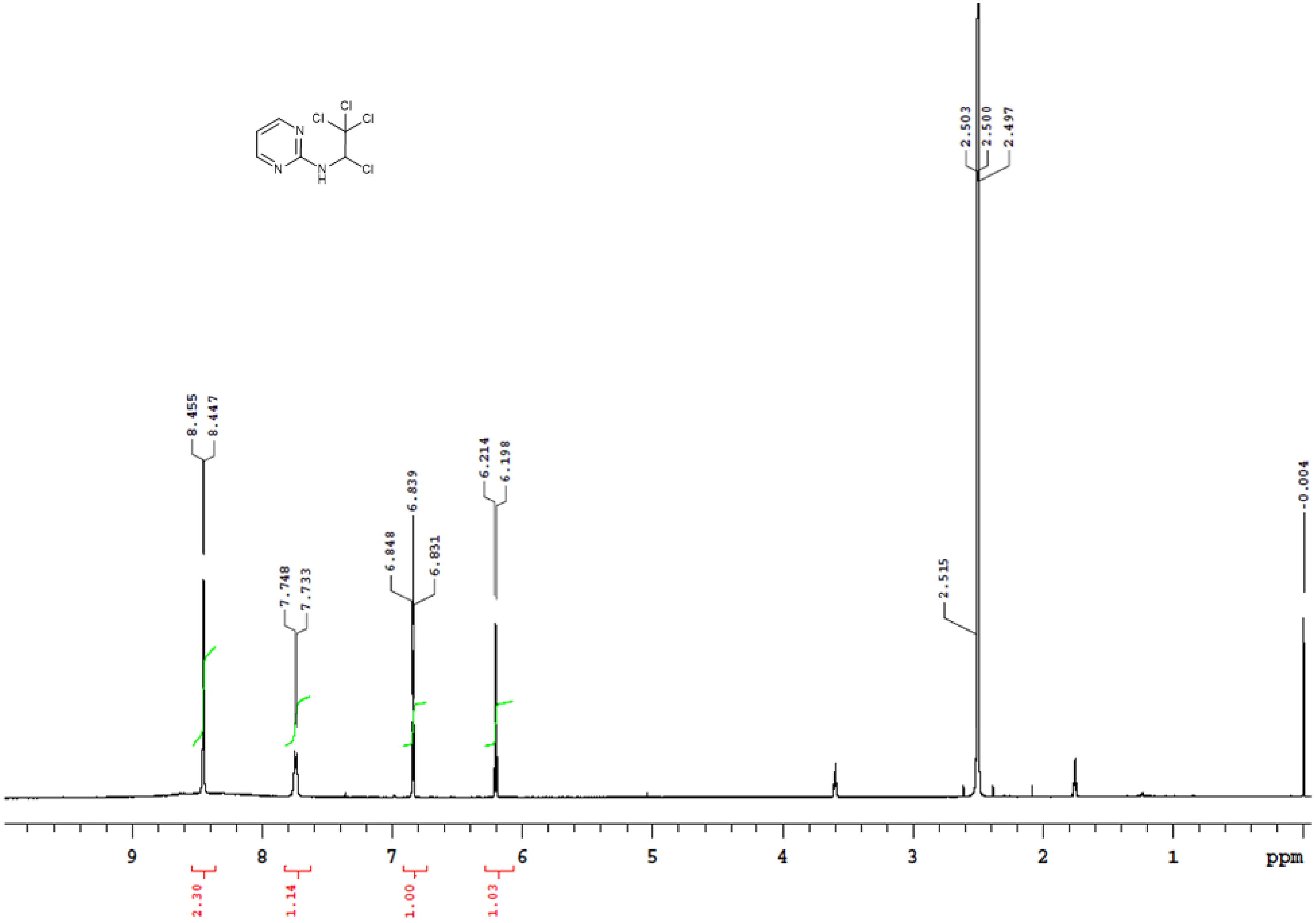

Figure S15. ^{1}H NMR spectrum of compound **8** in DMSO-d_6 (600 MHz, 25 °C)

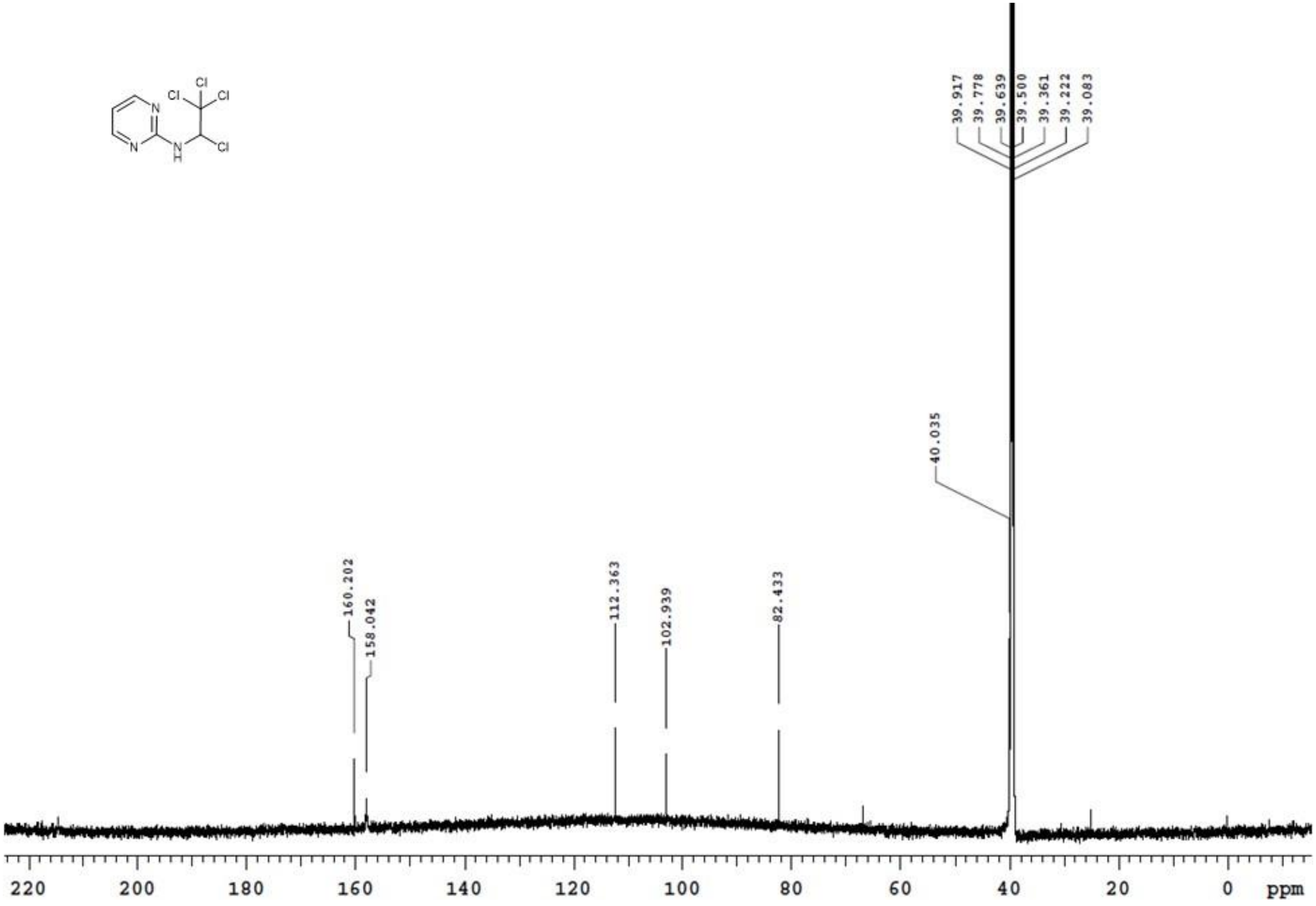

Figure S16. ^{13}C NMR spectrum of compound **8** in DMSO-d_6 (150 MHz, 25 °C)

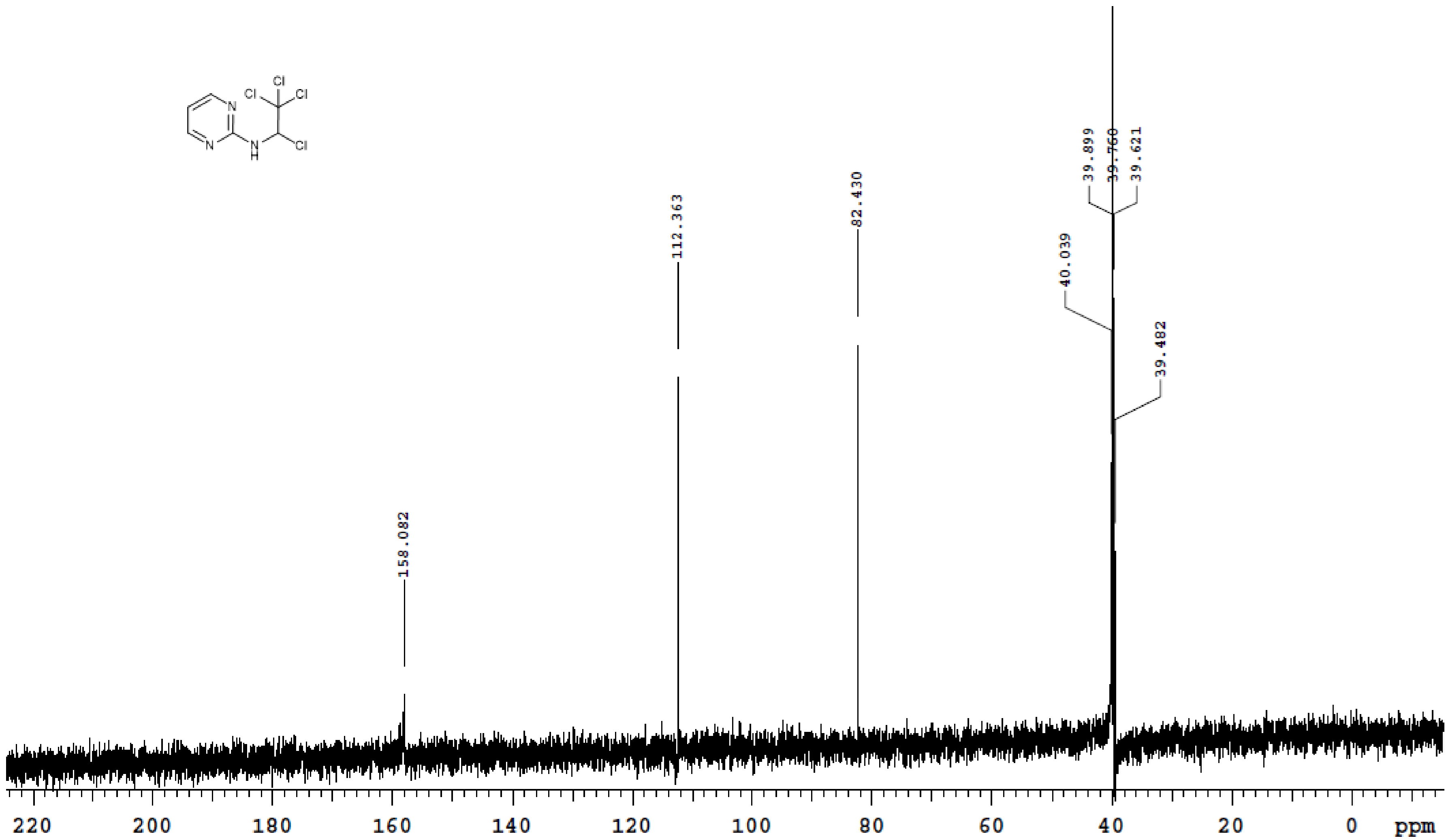

Figure S17. DEPT spectrum of compound **8** in DMSO-d_6 (150 MHz, 25 °C)

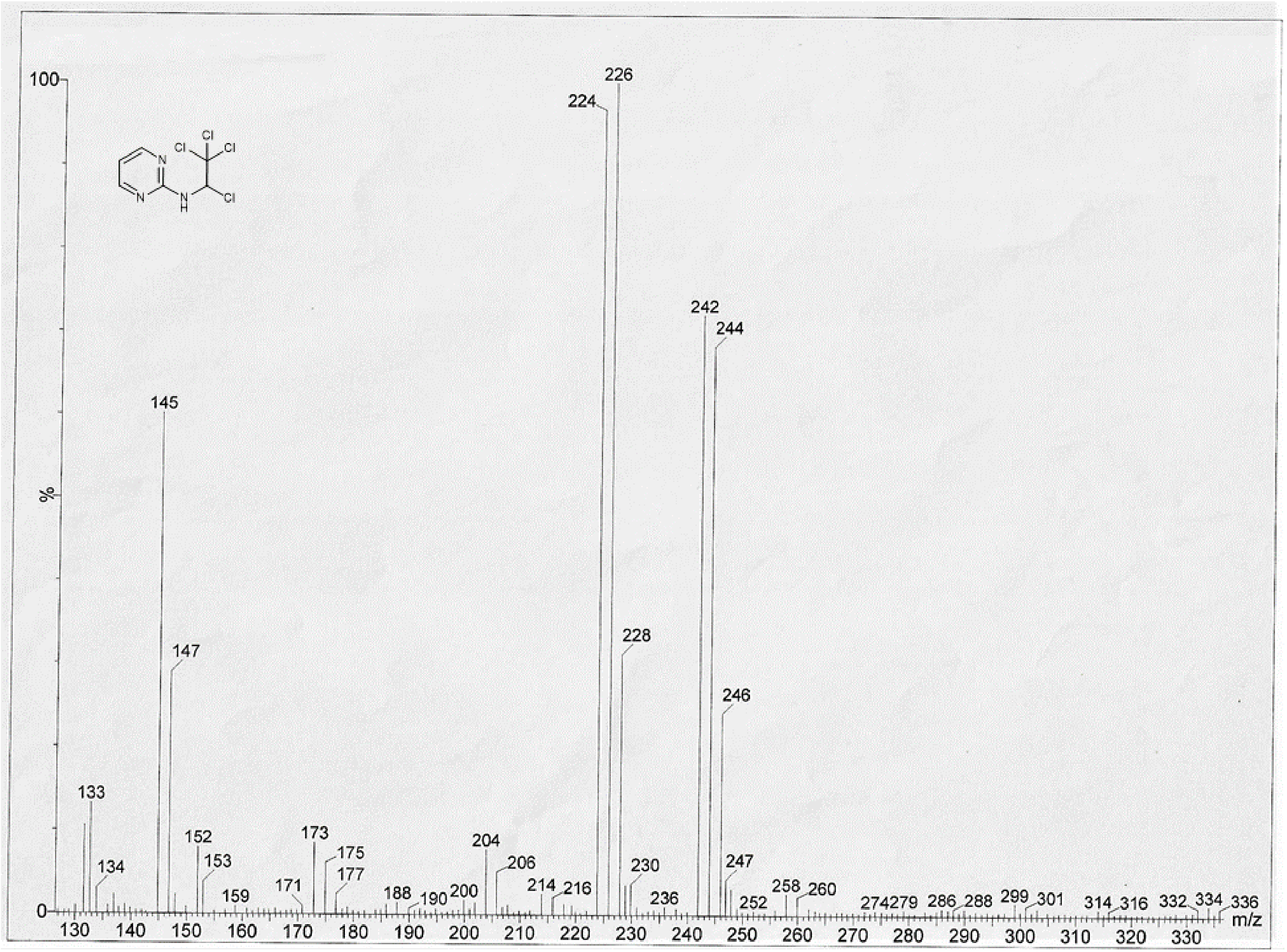

Figure S18. ESI-MS$^+$ spectrum of compound **8**.

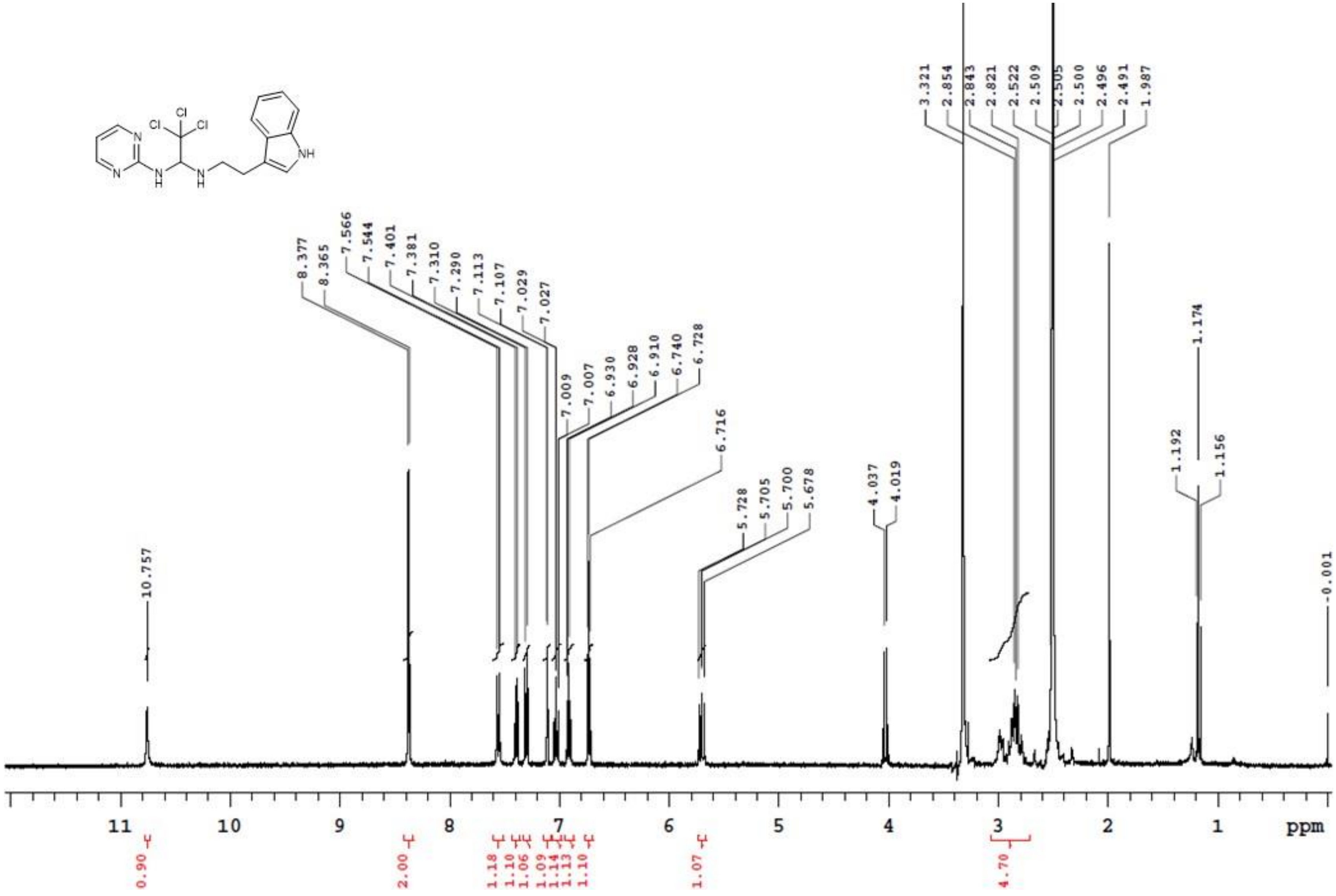

Figure S19. ^{1}H NMR spectrum of compound **9** in DMSO-d_6 (400 MHz, 25 °C)

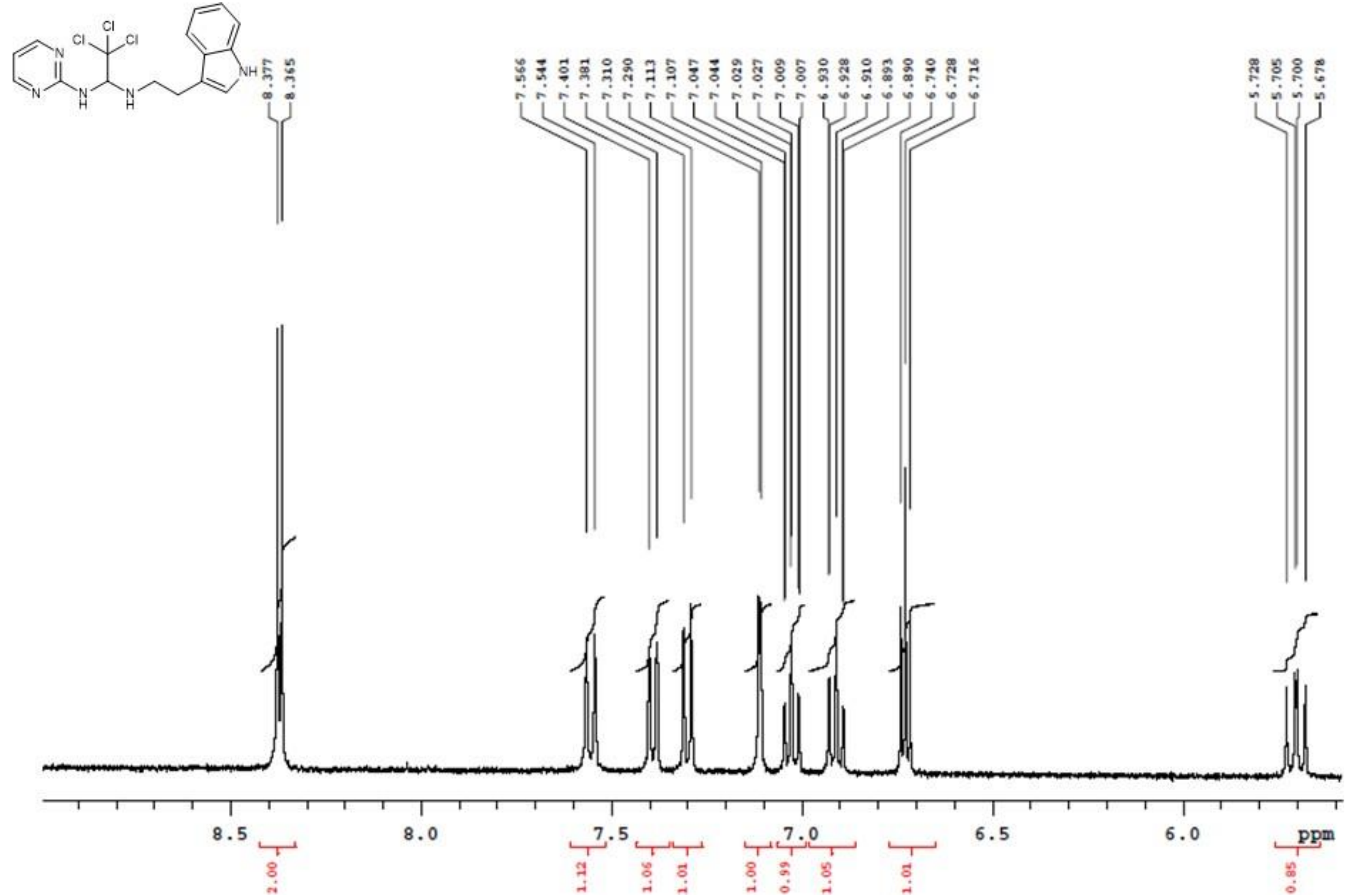

Figure S20. Expanded view of aromatic region of ^{1}H NMR spectrum of compound **9** in DMSO-d_6 (400 MHz, 25 °C)

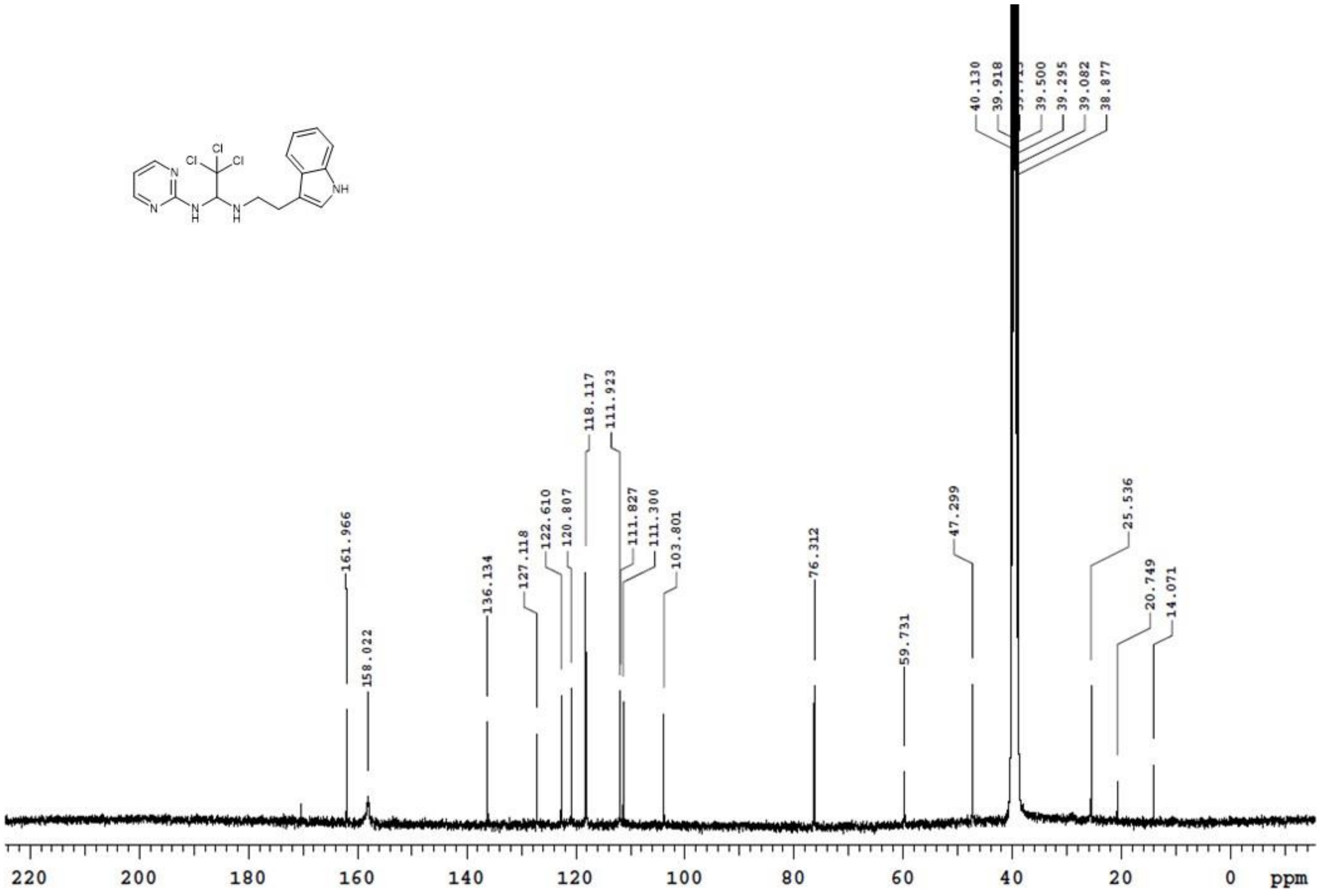

Figure S21. ^{13}C NMR spectrum of compound **9** in DMSO-d_6 (100 MHz, 25 °C)

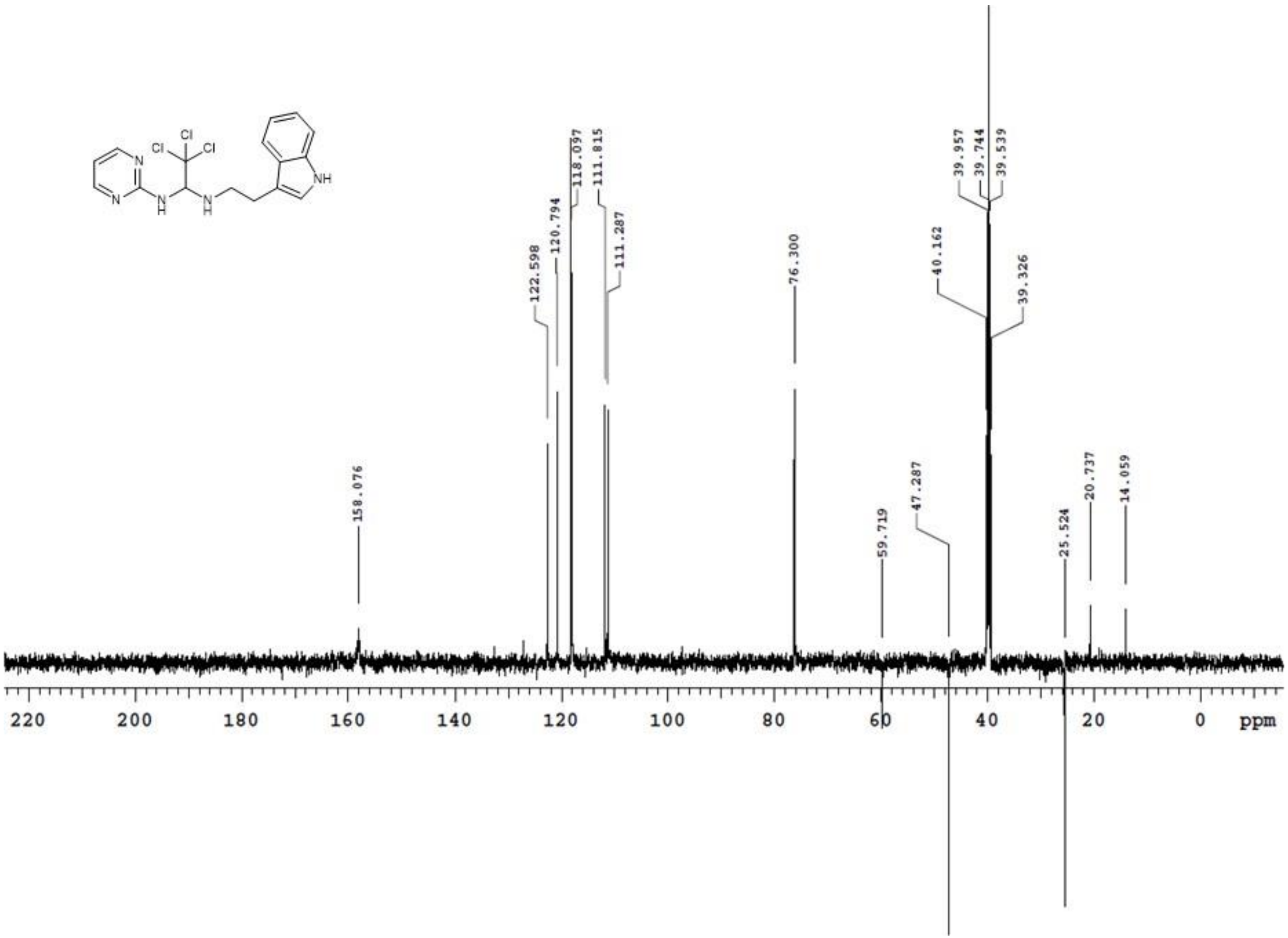

Figure S22. DEPT spectrum of compound **9** in DMSO-d_6 (100 MHz, 25 °C)

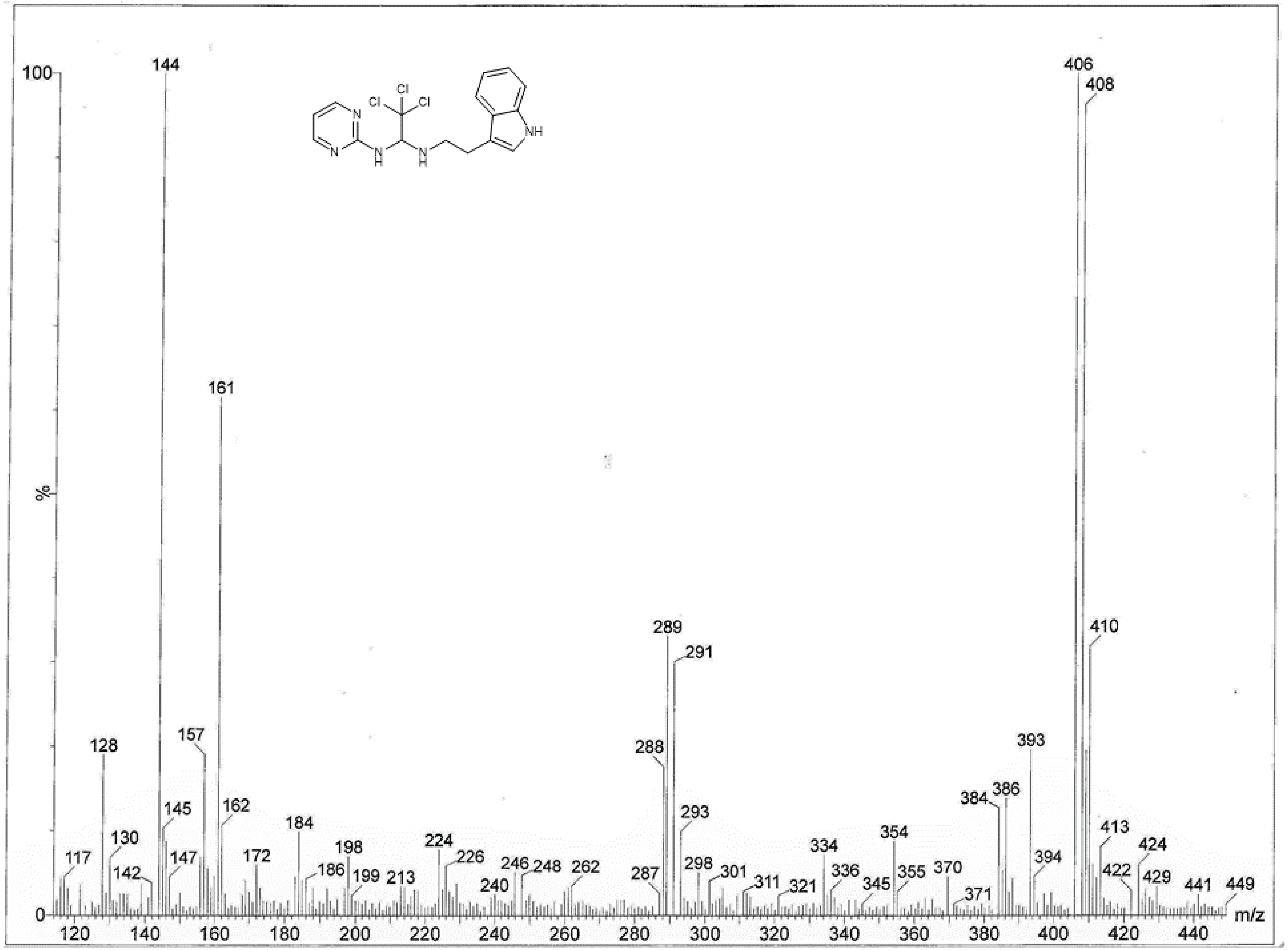

Figure S23. ESI-MS$^+$ spectrum of compound **9**.

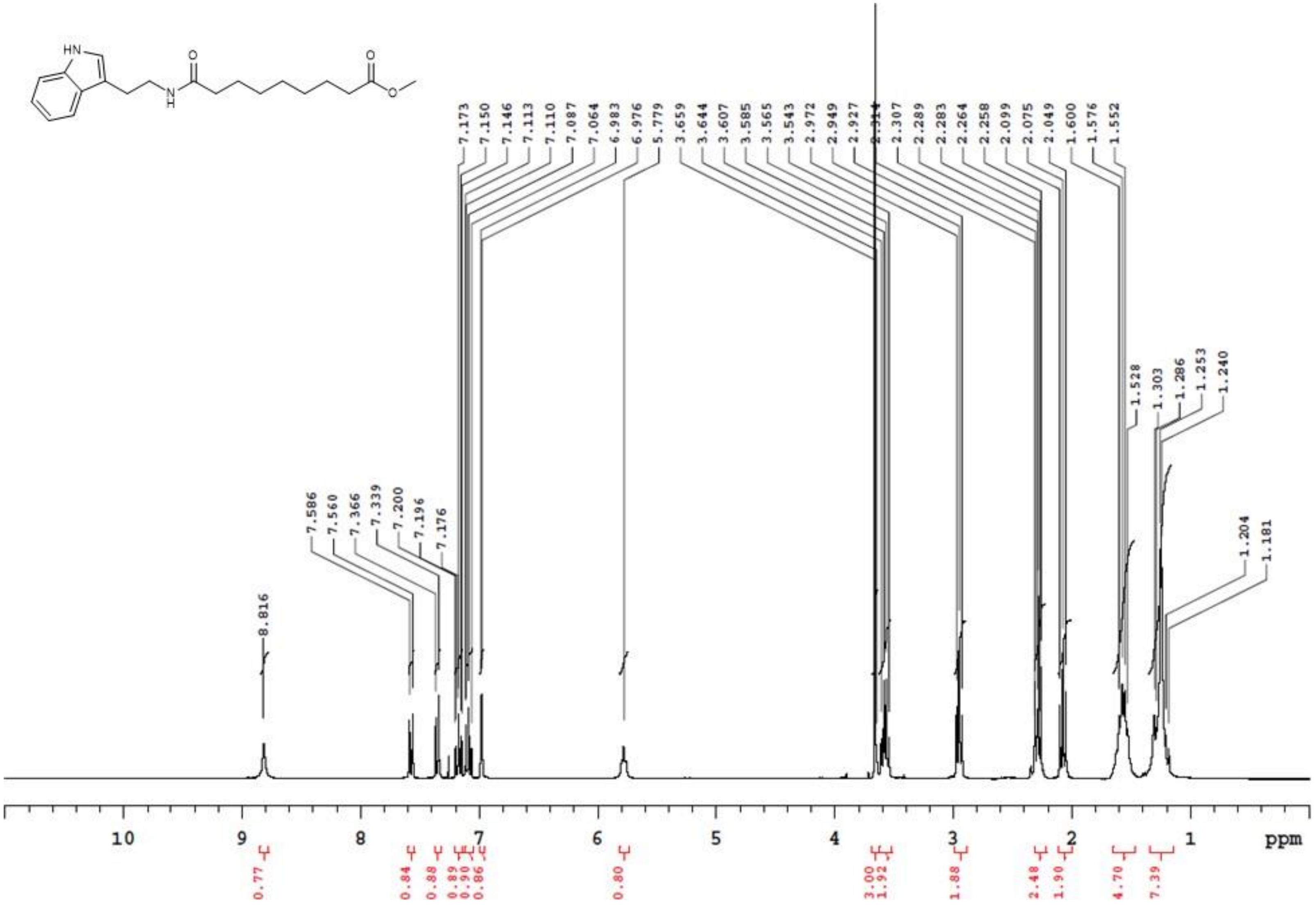

Figure S24. ^{1}H NMR spectrum of compound **13** in $CDCl_3$ (300 MHz, 25 °C)

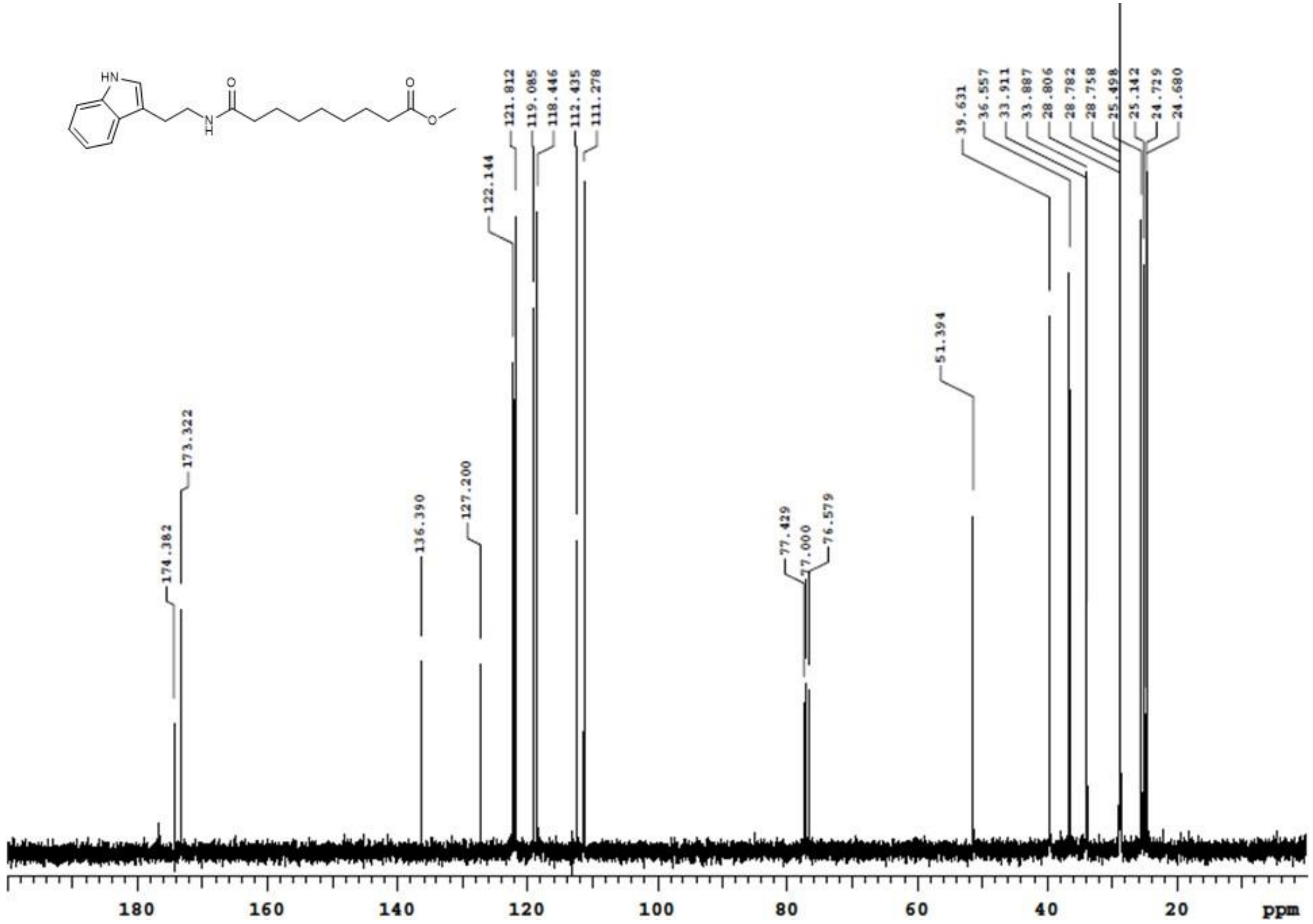

Figure S25. ^{13}C NMR spectrum of compound **13** in $CDCl_3$ (75 MHz, 25 °C)

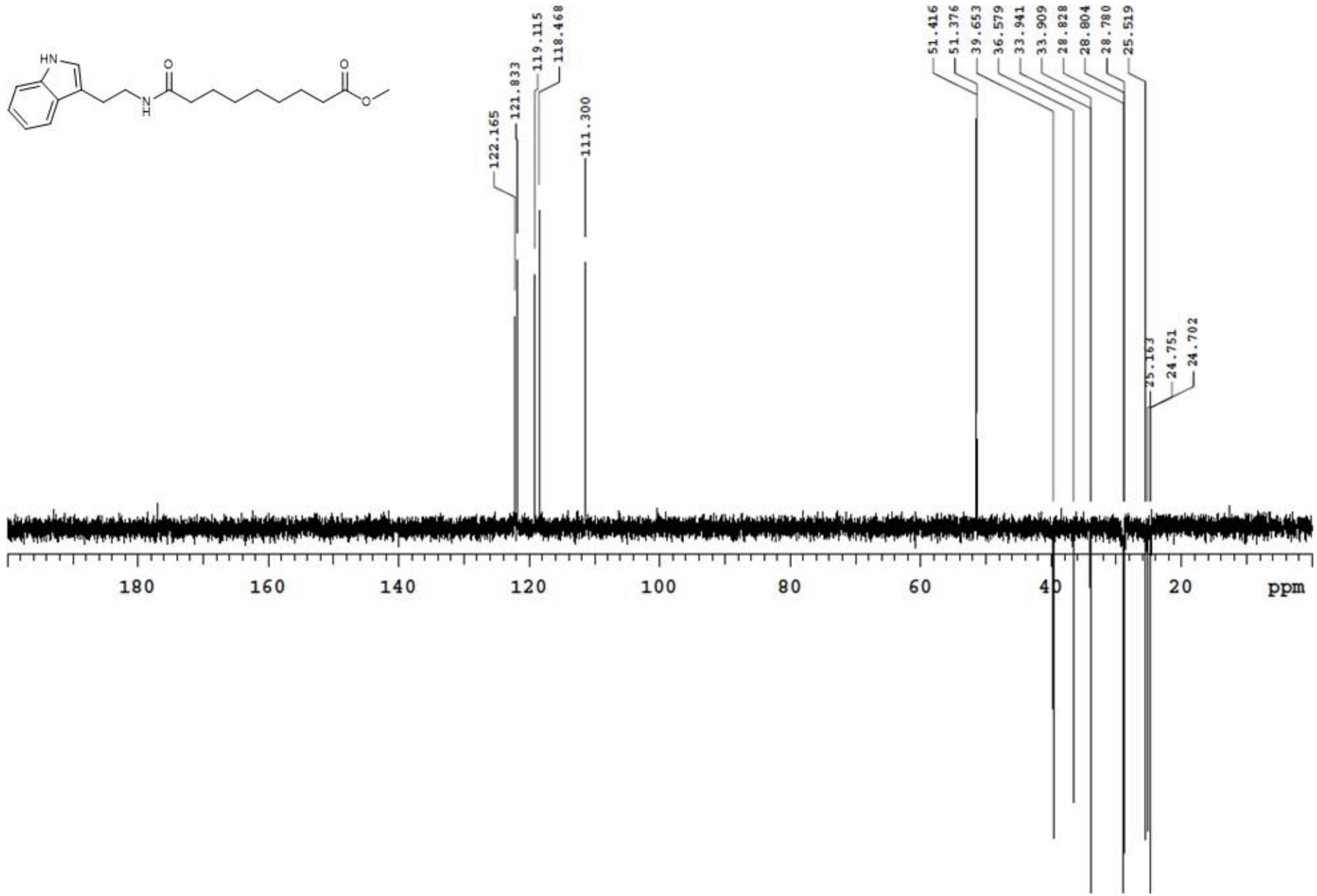

Figure S26. DEPT spectrum of compound **13** in $CDCl_3$ (75 MHz, 25 °C)

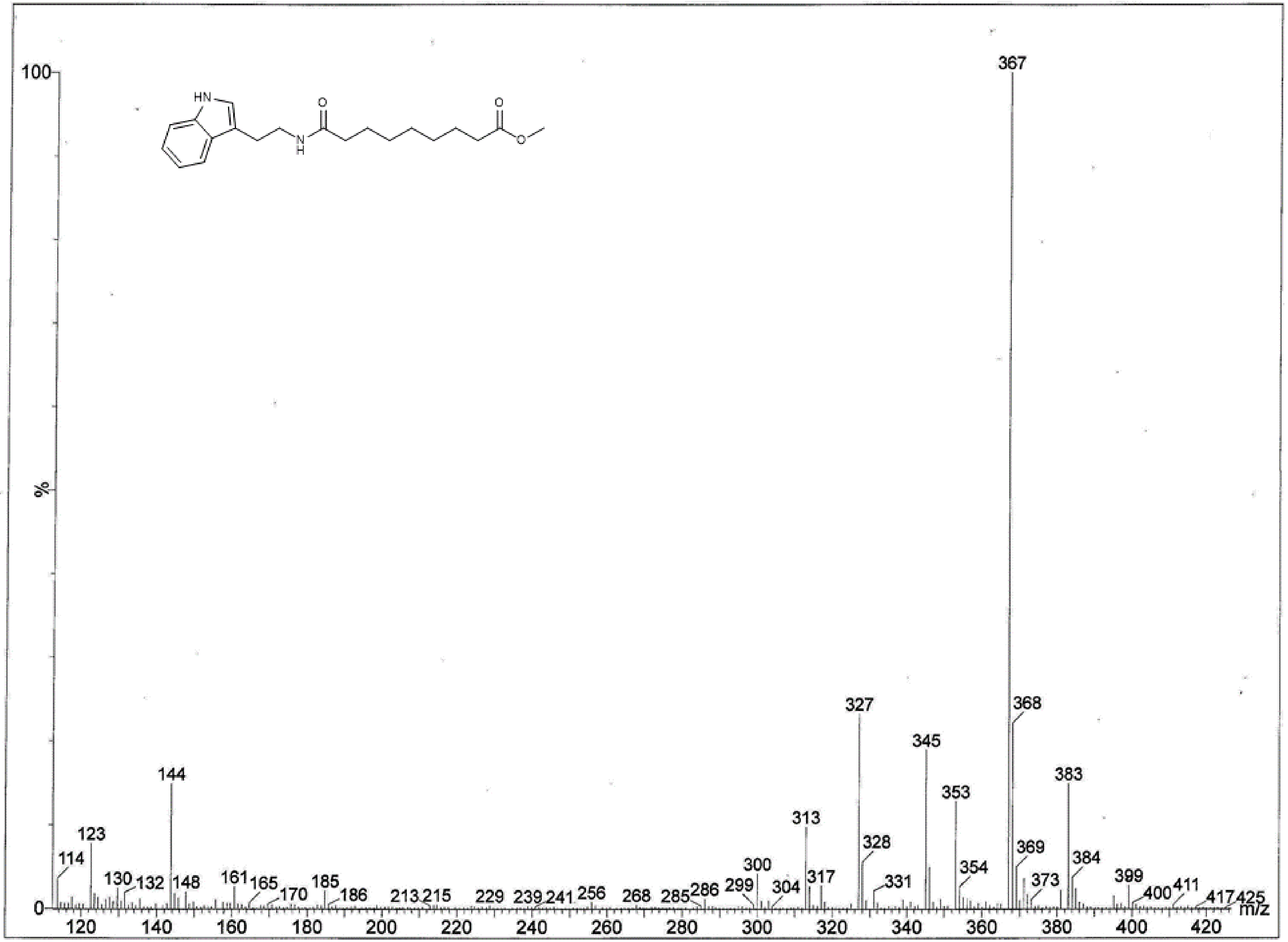

Figure S27. ESI-MS$^+$ spectrum of compound **13**.

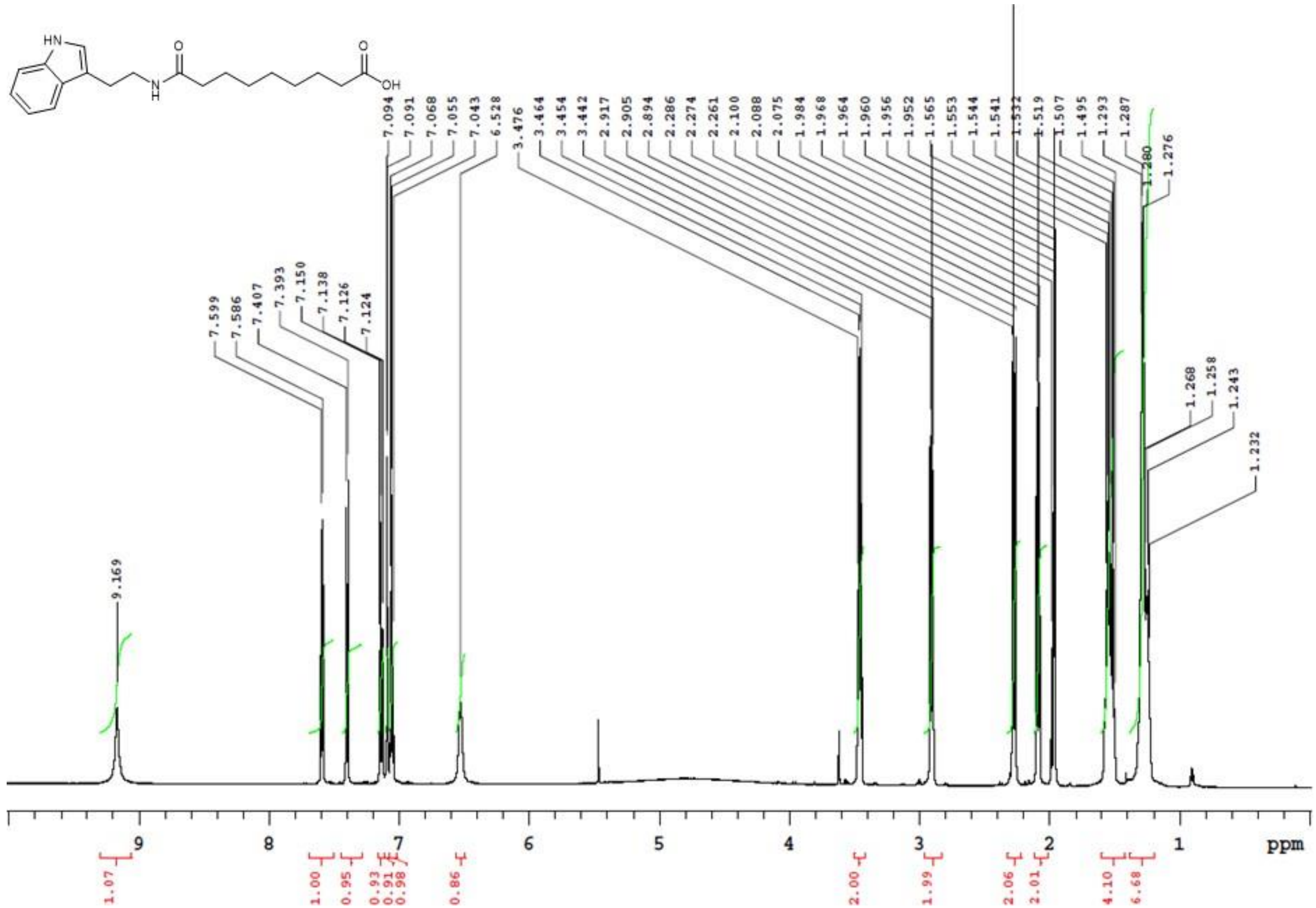

Figure S28. ^{1}H NMR spectrum of compound **14** in CD_3CN (600 MHz, 25 °C)

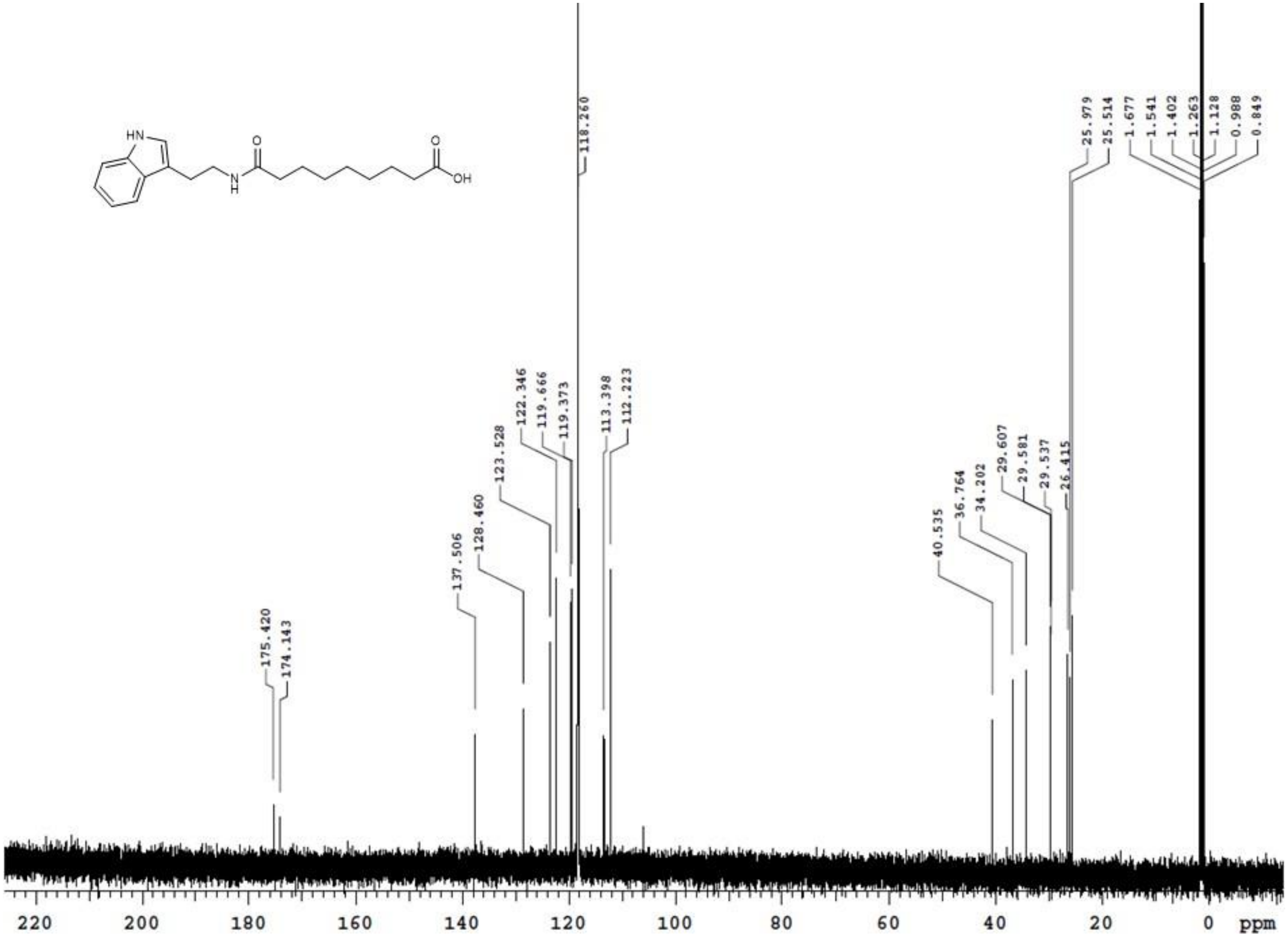

Figure S29. ^{13}C NMR spectrum of compound **14** in CD_3CN (150 MHz, 25 °C)

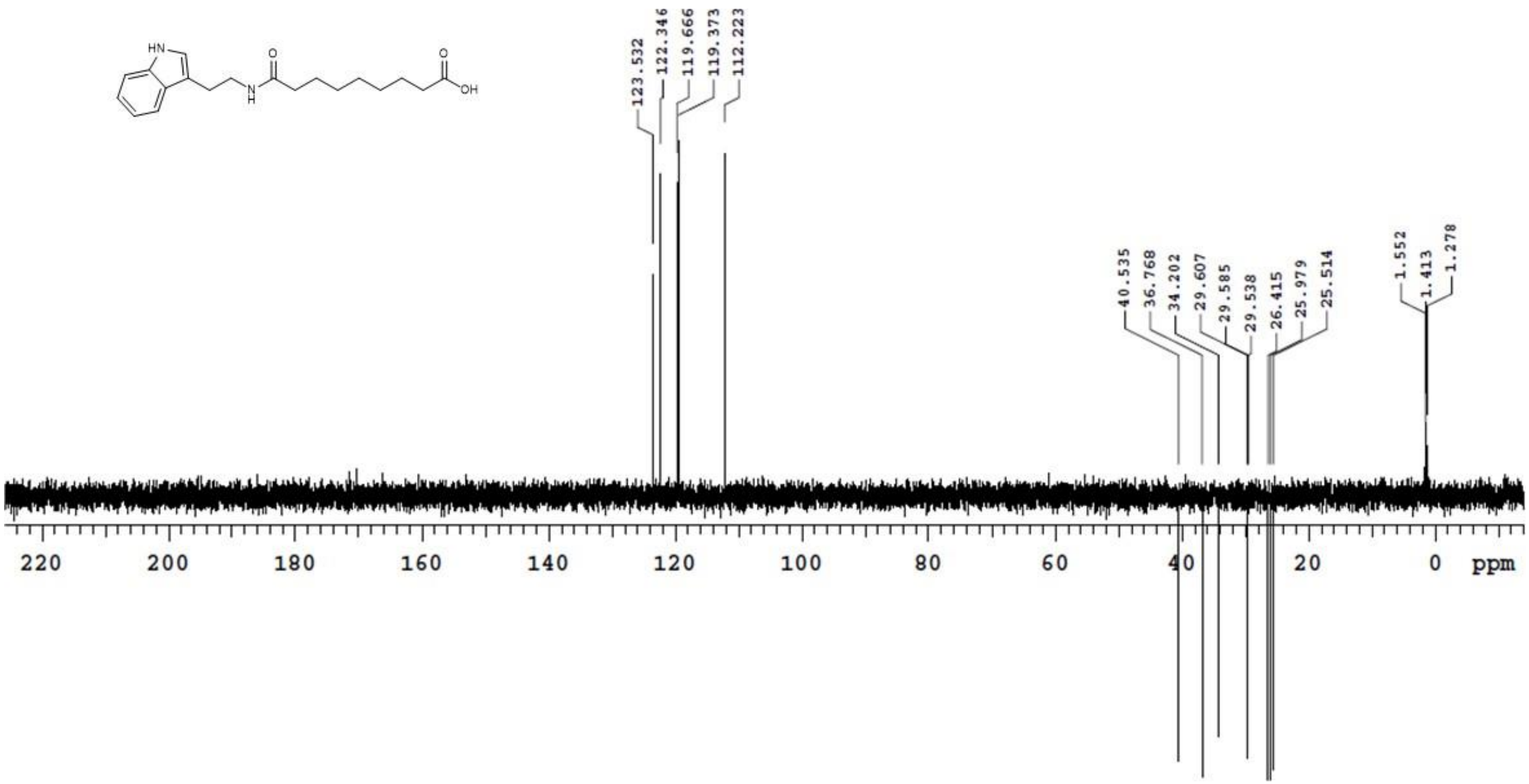

Figure S30. DEPT spectrum of compound **14** in CD_3CN (150 MHz, 25 °C)

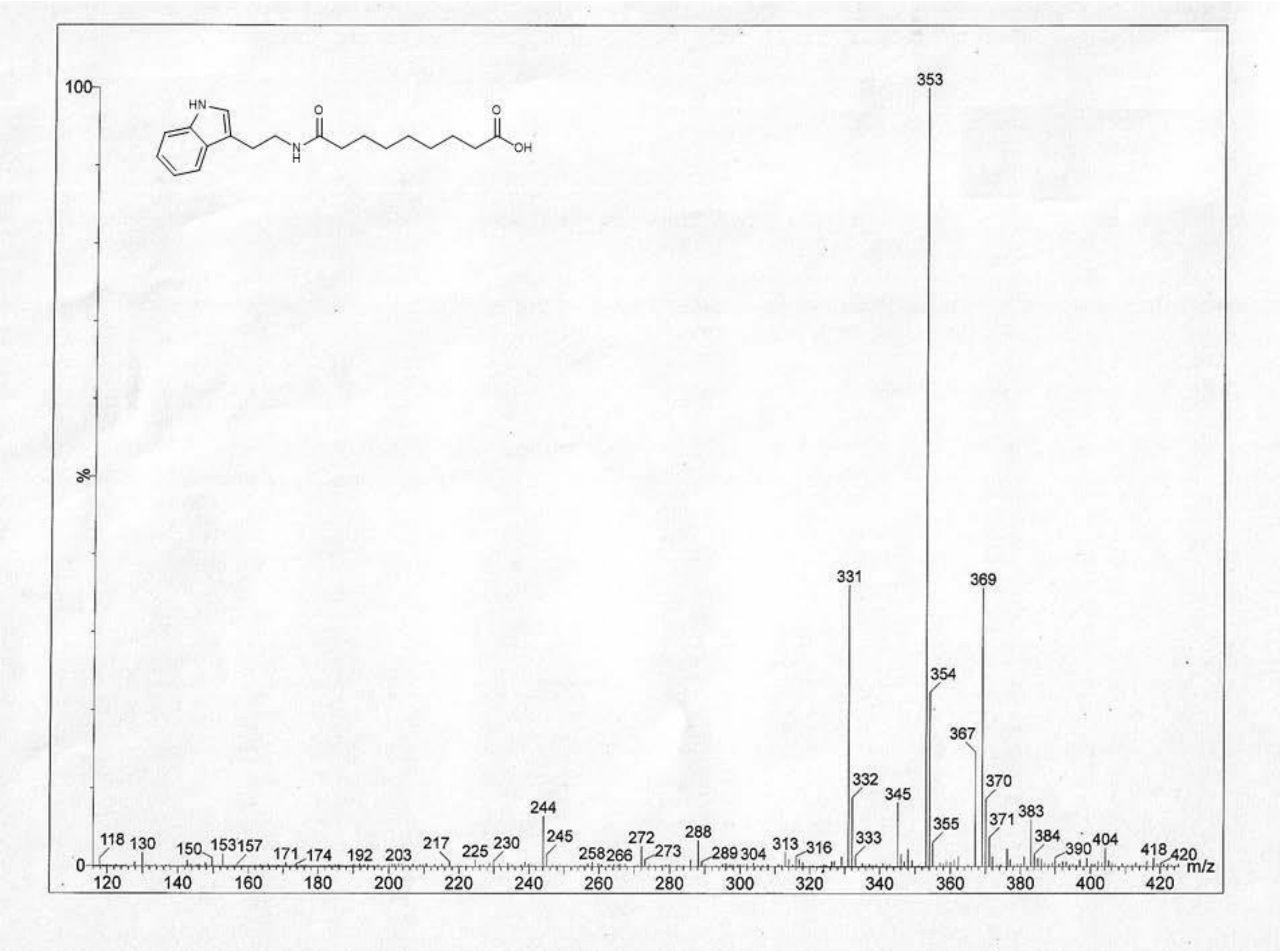

Figure S31. ESI-MS+ spectrum of compound **14**.
